# Blockade of PDGFRβ circumvents resistance to MEK-JAK inhibition via intratumoral CD8^+^ T-cells infiltration in triple-negative breast cancer

**DOI:** 10.1186/s13046-019-1075-5

**Published:** 2019-02-18

**Authors:** Murugan Kalimutho, Debottam Sinha, Deepak Mittal, Sriganesh Srihari, Devathri Nanayakkara, Shagufta Shafique, Prahlad Raninga, Purba Nag, Kate Parsons, Kum Kum Khanna

**Affiliations:** 1 Signal Transduction laboratory, QIMR Berghofer Medical Research Institute, 300 Herston Road, Herston, Brisbane, QLD 4006 Australia; 2 Immunology in Cancer and Infection Laboratory, QIMR Berghofer Medical Research Institute, 300 Herston Road, Herston, Brisbane, QLD 4006 Australia; 3 Institute for Molecular Bioscience, The University of Queensland, St Lucia, QLD 4072 Australia; 4 School of Environment and Science, Griffith University, Nathan, QLD 4111 Australia

**Keywords:** TNBC, PDGFRβ, JAK2, Resistance, CD8^+^ T cell

## Abstract

**Background:**

Despite the increasing progress in targeted and immune based-directed therapies for other solid organ malignancies, currently there is no targeted therapy available for TNBCs. A number of mechanisms have been reported both in pre-clinical and clinical settings that involve inherent, acquired and adaptive resistance to small molecule inhibitors. Here, we demonstrated a novel resistance mechanism in TNBC cells mediated by PDGFRβ in response to JAK2 inhibition.

**Methods:**

Multiple in vitro (subG1, western blotting, immunofluorescence, RT-PCR, Immunoprecipitation), in vivo and publically available datasets were used.

**Results:**

We showed that TNBC cells exposed to MEK1/2-JAK2 inhibitors exhibit resistant colonies in anchorage-independent growth assays. Moreover, cells treated with various small molecule inhibitors including JAK2 promote PDGFRβ upregulation. Using publically available databases, we showed that patients expressing high PDGFRβ or its ligand PDGFB exhibit poor relapse-free survival upon chemotherapeutic treatment. Mechanistically we found that JAK2 expression controls steady state levels of PDGFRβ. Thus, co-blockade of PDGFRβ with JAK2 and MEK1/2 inhibitors completely eradicated resistant colonies in vitro. We found that triple-combined treatment had a significant impact on CD44^+^/CD24^−^ stem-cell-like cells. Likewise, we found a significant tumor growth inhibition in vivo through intratumoral CD8^+^ T cells infiltration in a manner that is reversed by anti-CD8 antibody treatment.

**Conclusion:**

These findings reveal a novel regulatory role of JAK2-mediated PDGFRβ proteolysis and provide an example of a PDGFRβ-mediated resistance mechanism upon specific target inhibition in TNBC.

**Electronic supplementary material:**

The online version of this article (10.1186/s13046-019-1075-5) contains supplementary material, which is available to authorized users.

## Introduction

Basal-like, triple-negative breast cancer (TNBC) is a heterogeneous disease with no clinically approved targeted therapy [1]. Although chemotherapy is the mainstay treatment for TNBC, yet only 30% of the patients achieve a pathological complete response, while the remaining patients show recurrences as distant metastases. The failure to combat TNBC clinically has raised extensive efforts in identifying effective druggable molecular targets as well as combinatorial therapeutic strategies to treat these patients. Heterogeneity displayed by TNBC tumors promotes resistance, either innate or acquired to existing targeted agents. This possess substantial difficulty in obtaining a durable response as the tumor cells adapt to altered signaling networks through feedback mechanisms [2]. One of the well-characterized resistance mechanisms has been in response to Receptor tyrosine kinases (RTKs) or kinase inhibitors, which show redundancies with reprogramming of the kinome within the pathway or neighboring pathways to effectively bypass target inhibition [3, 4].

Recently, through Kinex™ antibody microarrays we have reported activation of diverse signaling networks in TNBC, dominated by signaling from receptor and non-receptor tyrosine kinases [5]. We found that HGF, EGF-MAPK, JAK-STAT3, VEGF, FGF, and TGFβ were amongst the most altered pathways in TNBC tumors. Inhibition of these pathways has been extensively investigated in TNBC therapy [1]; however, the effort to identify single pathway inhibition leads to disappointing results in clinics. The Extracellular Signal-Regulated Kinases 1 and 2 (*ERK1/2*) and the Janus Kinase 2 (*JAK2*) are the two major pathways that are significantly altered in TNBC pathogenesis [6–8]. Although *KRAS* mutations are not commonly found in breast cancer, the pathway seems to be hyperactive due to mutation in *NF1*, epigenetic silencing of *DUSP4* or other alternatives that lead to non-canonical MAPK activation [6, 7]. Likewise, JAK-STAT3 signaling is also hyperactivated in TNBC and is required for the maintenance of cancer stem cell-like population in basal-like breast cancers [8, 9]. Moreover, a recent study from the Arteaga laboratory has provided compelling evidence for JAK2-dependency in TNBC patients after chemotherapy treatment due to high rates of therapy-induced JAK2 amplification [10]. However, blockade of JAK1/2 using ruxolitinib in patients with refractory, metastatic TNBC demonstrated no clinical response despite evidence of on-target activity. This suggests rather complex mechanisms of resistance including intratumoral heterogeneity with clonal escape and immune evasion in clincial scenario [11]. Therefore, targeting these two pathways could offer a new avenue and useful strategy to treat TNBC.

The platelet derived growth factor ligands (PDGFs) and their cognate receptors (PDGFRs) play key roles in multiple signalling pathways including cell proliferation, migration and invasion, angiogenesis and metastasis. Overexpression of PDGF signalling has been observed in many human cancers including breast [12, 13]. Specifically, in breast cancer, PDGFRβ accumulation is seen in the stromal components [14, 15]. Its stromal expression is associated with high histopathological grade, high HER2 expression, ER negativity and shorter recurrence-free and cancer-specific survival [16]. PDGFRα and PDGFRβ have been shown to play a critical role in Foxq1-mediated epithelial–mesenchymal transition (EMT) and regulate cancer stemness and chemoresistance [17]. Notably, the autocrine PDGF/PDGFR loop facilitates TGF-β–induced EMT and metastasis through STAT1 [18].

In this report, we examine the response of targeting two parallel and overlapping pathways (MAPK and JAK/STAT) in TNBC. Through systematic analyses we showed a resistance mechanism mediated by PDGFRβ upregulation following JAK2 inhibition in TNBC cells. Co-treatment of TNBC cells with MEK1/2-JAK2 inhibitors failed to completely eradicate clonogenic growth under continuous drug exposure. Mechanistically, we found that JAK2 phosphorylates PDGFRβ at Y763 to fine-tune basal levels of PDGFRβ by regulating its proteolysis. Furthermore, we identified that the addition of a PDGFRβ inhibitor enhances the efficacy of combined MEK1/2 and JAK2 inhibition in vitro and significantly hampered TNBC syngeneic tumor growth in vivo through intratumoral CD8^+^ T cells infiltration.

## Method and materials

### Reagents

All small molecule inhibitors used in this study were purchased from Selleck Chemicals LLC (Houston, TX, USA) unless stated otherwise. Cycloheximide, MG132 and Pepstatin A were obtained from Sigma-Aldrich. Binimetinib (MEK162), Nilotinib and NVP-BSK805 were provided by Novartis (Switzerland) under a material transfer agreement. Small interfering RNAs (siRNAs) were purchased from Shanghai Gene Pharma (Shanghai, China). Lipofectamine®RNAiMAX and Lipofectamine® 3000 Reagents were purchased from Life Technologies, Carlsbad (CA, USA) and CellTiter 96® AQueous One Solution Cell Proliferation Assay from Promega Corporation, Fitchburg (WI, USA). Human Phospho-Receptor Tyrosine Kinase Array Kit was obtained from R&D Systems. Plasmids for STAT3 and JAK2 (wildtype and kinase dead) were a gift from Dr. Andrew Brooks, The University of Queensland Diamantina Institute, Australia. The HA-tagged PDGFRβ plasmid was a gift from Professor Jean-Baptiste Demoulin, Institut de Duve, Belgium. The GFP-PDGFRβ plasmid was a gift from Professor James Hagman, University of Colorado.

### Public databases

KMPlotter online tool (http://kmplot.com) was used to generate survival analysis in breast cancer patients [19]. cBioPortal online tool (http://www.cbioportal.org) was used to generate data related to mRNA expression [20, 21]. Genomics of Drug Sensitivity in Cancer (GDSC) database (www.cancerRxgene.org) was used to determine drug sensitivity [22, 23].

### Antibodies

List of antibodies used in this study are described in Additional file 1: Table S1.

### Cell culture

The breast cancer cell lines except 4T1.2 and HEK293T used in this study were purchased from the American Type Culture Collection (ATCC), otherwise stated in acknowledgment, cultured and maintained as per ATCC recommendations and as described previously [24]. All the cell lines were tested for Mycoplasma infection and authenticated using short tandem repeat (STR) profiling by scientific services at QIMR Berghofer Medical Research Institute.

### Plasmids transfection

Transient transfection in both SUM159PT and HEK293T were performed either using Lipofectamine® 3000 Reagents as per user’s manual or homemade Polyethyleneimine (PEI) reagent (for 1 μg DNA, 5 μL of 1 mg/ml PEI was used).

### Constructs and mutagenesis

Various PDGFRβ plasmids were generated by site-directed mutagenesis using QuikChange XL-II kit (Stratagene) according to the manufacturer’s instructions using primer sequences as shown in (Additional file 1: Table S1). Mutant constructs were verified by sanger sequencing.

### Small interfering RNAs and cell viability

Breast cancer cell lines were plated in 96-well plates at 5000–8000 cells/well followed by reverse transfection using 10 nM of siRNAs (Additional file 1: Table S1) for six days and cell viability were measured using CellTiter 96 Aqueous One Solution Cell Proliferation Assay Kit as described previously [25].

### 3D-spheroid culture

The 3D acini assay were performed using well established techniques as described previously [26].

### Colony formation assays

Drug treated cells (1*10^4^) were seeded on 12 well plates and incubated for additional 14 days to determine colony viability. The colonies were fixed with 0.05% crystal violet for 30 min, washed and representative images are shown in figures.

### Immunoblotting and immunoprecipitation

Immunoblotting was performed as described previously [27, 28]. For protein analysis, cells were lysed in Urea Buffer [8 M urea, 1% SDS, 100 mM NaCl, 10 mM Tris (pH 7.5)]. For immunoprecipitation, transfected cells were lysed in IP buffer [50 mM Tris-HCl pH 7.4, 300 mM NaCl, 5 mM EDTA, 1% Nonidet P40 and protease inhibitor cocktail] for 20 min on ice. Cell lysates were cleared by centrifugation and immunoprecipitated with the indicated antibodies for overnight at 4 °C. Protein complexes were collected after incubating for 2 h with Protein A/G-Sepharose Dynabeads (Invitrogen). Immunoprecipitates were washed thrice with PBS, eluted with 0.1 M Glycine (pH 2.5) and analyzed by western blot as described previously [27]. Immunodetections were performed using indicated primary antibodies (Additional file 1: Table S1) and horseradish peroxidase-conjugated anti-rabbit or mouse secondary antibodies (Amersham, GE Healthcare).

### Immunofluorescence

Cells were seeded and incubated overnight on 0.1% poly-l-lysine-coated coverslips that were fixed for 15 min in 4% paraformaldehyde in PBS, permeabilized in 0.5% Triton X-100-PBS for 15 min and blocked in 2% filtered bovine serum albumin (BSA). Primary antibodies were diluted in blocking solution and incubated with slides overnight at 4 °C. Alexafluor conjugated secondary antibodies were diluted 1/300 in blocking solution and stained for 45 min at 37 °C in humidifier chamber. Slides were washed, counterstained with DAPI (diluted 1/500 in blocking buffer, stock 1 mg/ml) and mounted in Prolong Gold. Slides were imaged using GE DeltaVision Deconvolution microscope and analyzed using Image J.

### Reverse transcriptase –quantitative PCR

RNA was extracted using RNeasy plus Mini Kit (Qiagen, Venlo, Limburg, Netherlands) and cDNA synthesized using the iScript™ cDNA Synthesis Kit (Bio-Rad) according to manufacturer’s instructions. RT-qPCR was performed on a CFX384 Touch™ Real-Time PCR Detection System (Bio-Rad, California, USA) using SYBR Green (Roche) and normalized against β-actin and HPRT1 as internal controls as described previously [24]. Primer sequences used in this study are described in Additional file 1: Table S1.

### Flow cytometry analysis of sub-G1

Flow cytometry analysis was performed to determine sub-G1 subpopulation following drug treatment, fixed in 70% ethanol overnight at 4 °C, washed, and stained with propidium iodide. Sub-G1 subpopulation was analyzed using MODFIT LT4.0 software Verity (Software House, Topsham, ME, USA).

### Flow cytometry analysis of CD24 and CD44 staining

Following 72 h of drug treatment, cells were trypsinized, washed twice with PBS and stained with fluorescence-conjugated -CD24 and -CD44 and their respective isotype controls (diluted in 1% serum) for 30 min on ice. Cells were washed in PBS twice and analyzed immediately. Data was acquired on a LSR IV Fortessa Flow Cytometer using the following configuration: PE (Ex 488 nm, 570 nm LP mirror, 586/15 nm BP filter) and APCH7 (Ex 640 nm, 750 nm LP mirror, 680/60 nm BP filter). Debris and dead cells were excluded from analysis based on forward and side scatter.

### Flow cytometry analysis of immune cells

Spleens and tumors were mashed, filtered with 70 μm cell strainer, and washed with 1% FBS-PBS. Red blood cells were lysed with ACK buffer incubation for 1 min at RT. Single-cell suspensions were incubated for 15 min in 1% FBS-PBS and stained with the following fluorescence-conjugated antibodies (Additional file 1: Table S1). Samples were acquired on a LSR IV Fortessa Flow Cytometer (BD Biosciences). Data were analyzed on FlowJo V10 (Treestar).

### In vivo xenografts

All experiments were accordance to the guidelines of the QIMR Berghofer Medical Research Institute Animal Ethics Committee and as described previously [27]. Briefly, 5–6 weeks old female Balb/C Nude or BALB/C mice were used in this study. All mice were housed in standard condition with a 12 h light/dark cycle and free access to food and water. For mammary fat pad injections, 3.0 × 10^6^ human MDA-MB-231 cells were prepared in 50% Matrigel (BD, Biosciences, Bedford, USA)/PBS and injected into the right 4th inguinal mammary fat pad of 6 week old Balb/C nude mice. For mouse  4T1.2 tumor cells injection in BALB/C mice, 0.1 × 10^6^ were prepared in PBS. Tumor growth was measured thrice weekly by caliper measurements. To calculate tumor area the following formula was used: tumor area = B*S where B = largest tumor measurement and S = the smallest, based on two-dimensional caliper measurements.

### Predicting response to Ruxolitinib in TCGA patients

We developed a machine-learning (ML) model to predict the clinical response to JAK2 inhibition in TCGA patients by training a multinomial logistic regression classifier on response (IC50 values) to the JAK2-inhibitor Ruxolitinib on 982 cell lines from the Genomics of Drug Sensitivity in Cancer (GDSC) database (www.cancerRxgene.org). The IC50 values were distributed as: Min screening conc: 0.0204, Max screening conc: 5.24, and Geometric mean: 53.2 (Fig. 2a). The cell-lines were annotated as Sensitive (IC50 ≤ 5.24; 23 cell lines), Moderately sensitive (5.24 < IC50 ≤ 53.2, 369 cell lines) and Resistant (IC50 > 53.2; 590 cell lines). We trained the classifier to learn this response labels as a function of the Z-score normalized mRNA (RNAseq) expression values of genes belonging to the JAK/STAT signalling pathway (139 genes, KEGG database) for the cell lines (downloaded from cBioPortal) [21]. Five-fold (80–20%) cross-validation of the classifier on the cell line data gave a maximum accuracy of 71% and on average 63%. We then trained the classifier on 100% of cell-line data and applied it to predict Ruxolitinib response on 1093 breast carcinoma patient data (219 TNBC or ER-neg/PR-neg patients) from TCGA (predicted sensitive: 85 patients, moderate: 434 patients, and resistant: 574 patients). We validated the predictions on TCGA by plotting survival curves (overall survival and disease-free survival) of the Sensitive, Moderate and Resistant subgroups of patients, with the premise that if resistance to Ruxolitinib indeed impact clinical outcomes then the patients predicted to be in these three subgroups are likely to show distinct clinical outcomes.

#### Computing enrichment for Ruxolitinib sensitivity among ER-/PR- patients

We computed the enrichment for Ruxolitinib sensitive and Ruxolitinib resistance within the ER-/PR-patients using a hypergeometric test, as follows.

If X is a random variable that follows the hypergeometric distribution and measures the number of successes (sensitive or resistant patients) in ER-/PR- patients, then the enrichment *p*-value for sensitive/resistance among ER-/PR-patients is:

P (*X* = *k*) = Σ_0 ≤ *k* ≤ *|C|*_ (*|C|* choose *k*) ((*N*-*|C|*) choose (*n*-*k*))/(*N* choose *n*),where.

*k* = number of sensitive/resistant patients among ER-/PR-patients,

*n* = number of *sensitive/resistant patients* in the entire TCGA dataset, and.

*N* = the population size (TCGA dataset, 1093 patients).

We consider *P* ≤ 0.05 as statistically significant enrichment for sensitive/resistance among ER-/PR- patients.

### Statistical analysis

All comparisons between samples were evaluated using the two-tailed non-parametric Mann-Whitney test, one-way or two-way ANOVA with Bonferroni post hoc testing unless otherwise stated in figure legends using GraphPad Prism v7.0 (GraphPad Software, LaJolla, CA, USA). Where applicable, statistical significance is denoted by * for *P* ≤ 0.05, ** for *P* ≤ 0.01, *** for *P* ≤ 0.001, and **** for *P* ≤ 0.0001 and n.s. = not significant. Data are expressed as mean ± Standard error (SEM).

## Results

### MEK1/2 and JAK2 inhibition-mediated resistance in triple-negative breast cancer

As both KRAS-dependent ERK1/2-MYC and IL6/8-dependent JAK-STATs signaling have been shown to be significantly altered in TNBC [29–32], we asked if co-inhibition of these signaling axes would synergistically kill TNBC. To test the alteration of these pathways in individual breast cancer patients in a subtype specific context, we calculated pathway dysregulation score using Pathifier [33]. TNBC patients showed higher dysregulation scores for both pathways when compared to ER^+^ patients (Additional file 2: Figure S1A). To further elucidate the pathway dependency in a panel of breast cancer cell lines, we depleted both JAK1 and JAK2 kinases (upstream regulators of STAT proteins) and found that TNBC cell lines are more likely to be dependent on JAK2 than JAK1 for survival (Additional file 2: Figure S1B, C). However, surprisingly moderate growth inhibition was observed following STAT3 silencing (Additional file 2: Figure S1B, C). Interestingly, despite the absence of prevalent mutations of KRAS in breast cancer patients [34], silencing of KRAS significantly inhibited cell viability in most TNBC lines compared to non-TNBC lines, consistent with pathway activation reported in TNBC [6, 7] (Additional file 2: Figure S1B, C).

**Fig. 1:**
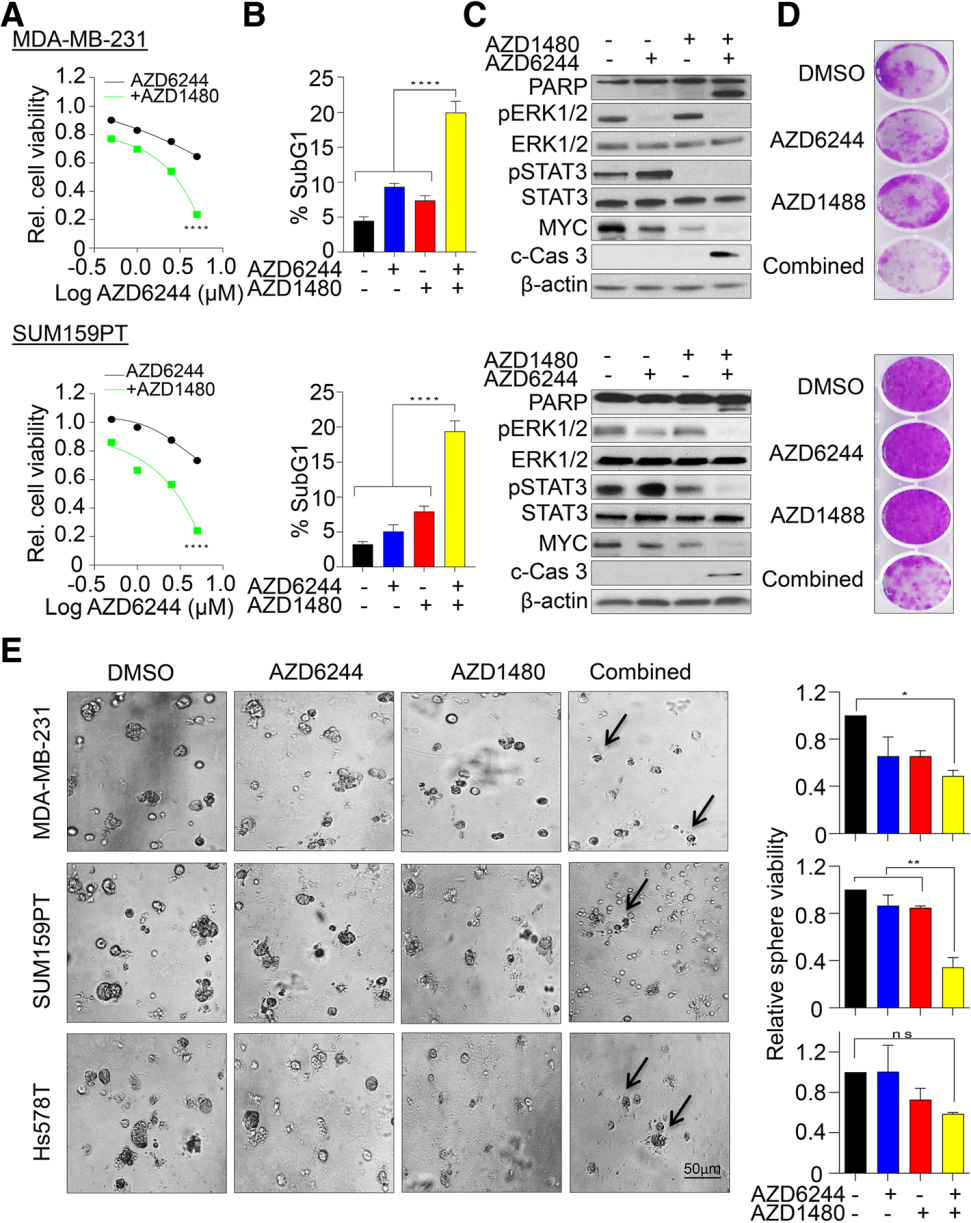
*MEK1/2 and JAK2 inhibition-mediated resistance in TNBC cells*. **a** MDA-MB-231 (upper panel) and SUM159PT (lower panel) cells were exposed to different concentrations of MEK1/2 inhibitor (AZD6244) alone or in combination with JAK2 inhibitor (AZD1480 2.5 μM) and cell viability was determined after 6 days using MTS assays. The dose-response curve was generated by calculating cell viability relative to untreated control and plotted against drug concentration, *n* = 3 with SEM (****p < 0.0001). **b** Percentage of sub-G1 population identified using propidium iodide staining and quantified by FACS following single and combination treatment with AZD6244 (1 μM) and AZD1480 (2.5 μM) inhibitors after 72 h, *n* = 3 with SEM (****p < 0.0001). **c** Immunoblot analysis of both SUM159PT and MDA-MB-231 cell lines treated with single and combination treatments after 48 h and levels of indicated proteins were determined. **d** Representative images of colony forming capacity after single and combination treatment at 14 day determined using crystal violet staining. **e** Left, representative phase-contrast images of MDA-MB-231, SUM159PT and HS578T cells grown on Matrigel for 14 days. Cells were treated with indicated drugs after 2 days of seeding. Right, Relative sphere viability determined using MTS assay. *n* = 2 with SEM (*p < 0.05, **p < 0.01, ns: not significant)

In a view of designing more effective therapeutic strategies, we treated various TNBC cell lines with a selective MEK1/2 (AZD6244) and JAK2 (AZD1480) inhibitors and assessed their cell viability over a period of 6 days. We found that both single agents have less anti-proliferative potency compared to combination treatment in TNBC cell lines (Fig. 1a, Additional file 3: Figure S2A). Moreover, this combined treatment significantly induced apoptosis, evaluated by the accumulation of a propidium iodide stained-Sub-G1 apoptotic fraction (Fig. 1b) in addition to expression of cleaved PARP and Caspase 3 (Fig. 1c, Additional file 3: Figure S2B). Consistent with a previous report in colorectal cancer lines [35], we observed a marked increase in phosphorylated STAT3 after MEK1/2 inhibition in breast cancer lines, suggesting an acute rewiring of compensatory pathway upon MEK1/2 inhibition (Fig. 1c). To exclude cell line specific effects, we co-treated additional four breast cancer cell lines with both AZD6244 and AZD1480 inhibitors and found that co-inhibition induced apoptosis in most of the TNBC lines (Additional file 3: Figure S2C) but not in the non-TNBC line, MDA-MB-453 (Additional file 3: Figure S2D). Moreover, suppression of long-term colony forming capacity upon combined inhibition in both 2D and 3D spheroid cultures further elucidated an emergence of residual resistant sub-clones (Fig. 1d, e). Collectively, this data suggested that even though the individual or combination inhibition of multiple pathways appears to be a viable therapeutics strategy in TNBC treatment, yet rewiring of compensatory pathways still pose a significant challenge in controlling the outgrowth of TNBC cells.

### JAK2 inhibition mediated PDGFRβ accumulation

**Fig. 2:**
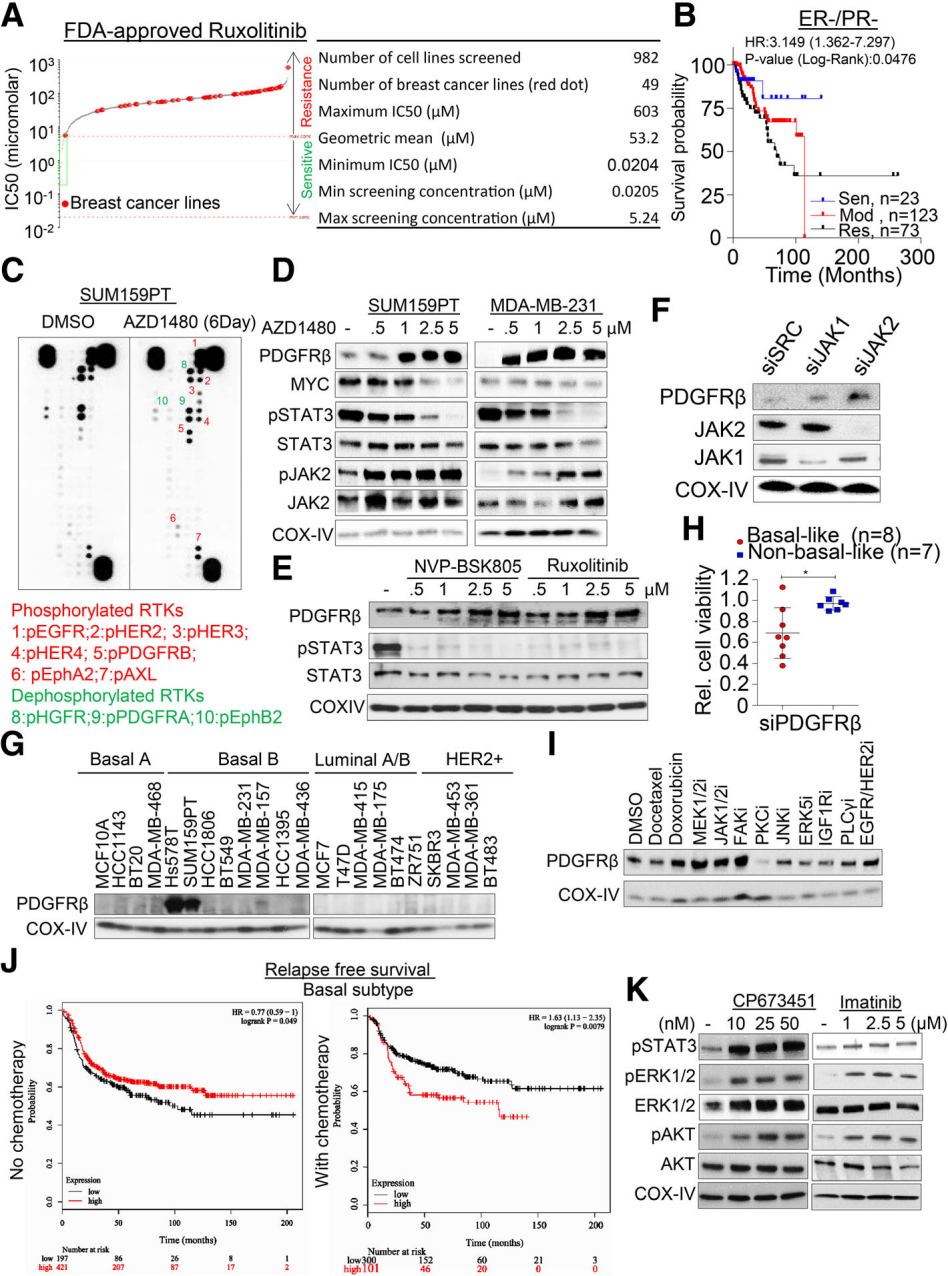
*JAK2i-mediated PDGFRβ accumulation in TNBC cells*. **a** IC50 scatter plot of a large panel of cancer cell lines (*n* = 982) for Ruxolitinib derived from genomics of drug sensitivity in cancer database (http://www.cancerrxgene.org). Table shows detailed analysis of geometric mean wherein the number of breast cancer cell lines is shown in red dots. **b** Prediction of Kaplan-Meier survival analysis in TCGA patients using data derived from cell line treated with Ruxolitinib and computed based on machine-learning (ML) model. See methodology for details of the analysis. **c** SUM159PT cells were continuously treated with 5 μM AZD1480 for 6 days and analyzed using receptor tyrosine kinase array. **d** Dose-dependent PDGFRβ accumulation in response to AZD1480. SUM159PT and MDA-MB-231 cells were treated with different concentrations of AZD1480 for 24 h and indicated proteins were determined by western blot. **e** SUM159PT cells were treated with different concentrations of two JAK2-specific inhibitors and PDGFRβ levels were determined by western blot. **f** SUM159PT cell were reversed transfected with 10 nM of JAK1 and JAK2 pool siRNAs for 48 h and PDGFRβ levels were determined by western blot. **g** Western blot analysis of PDGFRβ protein levels in a panel of human breast cancer lines (*n* = 22). Cell lines were divided based on their respective subtypes.**h** A panel of selected breast cancer and near-normal cell lines were reverse-transfected with 10 nM PDGFRβ siRNA and cell viability was determined after 6 days. Cell viability relative to its own respective control transfected with scramble siRNA was calculated, *n* = 2–3 with SEM (*p < 0.05). **i** SUM159PT cells were treated with multiple small molecules inhibitors against several signaling pathways for 24 h and PDGFRβ levels were determined by western blot. **j** Kaplan-Meier survival analysis of the relationship between *PDGFRβ* mRNA expression and clinical outcomes in breast cancer patients treated with or without chemotherapy using the KMplotter dataset (http://kmplot.com/). *PDGFRβ* expression stratified on relapse free survival. **k** SUM159PT cells were treated with different concentration of PDGFRβ inhibitors for 24 h and the levels of growth and survival-related proteins were determined using western blot

Resistance to MEK1/2 inhibition has been well documented in several types of human cancers [36]. Therefore, to investigate the resistance mechanisms mediated by JAK2 inhibition in our setting, we developed a machine-learning (ML) model to predict the clinical response to JAK2 inhibition in breast cancer patients. We trained our model on JAK2-inhibitor Ruxolitinib response (IC50: Min screening conc: 0.0205, Max screening conc: 5.24, Geometric mean: 53.2) using 982 tissue-specific cancer cell lines from the Genomics of Drug Sensitivity in Cancer (GDSC) database (www.cancerRxgene.org) [22] (Fig. 2a). About 40% of cell lines showed moderate (5.24 < IC50 ≤ 53.2; 369 cell lines) to high (IC50 ≤ 5.24; 23 cell lines) sensitivity to Ruxolitinib, while the remaining (~ 60%) cell lines were considered resistant (IC50 > 53.2; 590 cell lines) (Fig. 2a). We trained a multinomial (multiclass) logistic regression classifier to learn the cell-line mediated response to Ruxolitinib and used the data as a function of the Z-score normalized mRNA (RNAseq) expression values of genes in the JAK/STAT signaling pathway (139 genes, KEGG database). Five-fold (80–20%) cross-validation of the classifier gave a maximum accuracy of 71% and on average 63%. We then trained the classifier on 100% of cell-line data and applied it to predict Ruxolitinib response (predicted sensitive: 85 patients, moderate: 434 patients, and resistant: 574 patients) on 1093 breast carcinoma patient data (219 ER-neg/PR-neg patients) from TCGA cohort. Since the exact matching response data (response to Ruxolitinib treatment) was not available for validation, we analyzed the results of our classifier in two ways: (i) the three predicted subgroups showed significantly distinct (logrank-test *p* = 0.0476) disease-free/relapse-free survival outcomes in ER-neg/PR-neg patients (Fig. 2b) compared to ER-pos/PR-pos patients (Additional file 4: Figure S3A); (ii) the ER-neg, PR-neg patients were significantly less enriched for sensitive (hypergeometric-test *p* = 3.66E-10) response compared to being moderate or resistant to Ruxolitinib. These analyses indicate that nonresponse to JAK/STAT inhibition could be associated with the tendency to relapse (resistant) in majority of ER-neg, PR-neg breast cancer patients compared to ER-pos/PR-pos patients (Additional file 4: Figure S3B).

To further determine JAK2 inhibition-mediated resistance in vitro, SUM159PT cells were exposed to 5 μM of JAK-pathway inhibitor AZD1480 for 6 days and then subjected to a phospho-receptor tyrosine kinase array screen (Fig. 2c). We identified that the EGFR family members (HER3 and HER4) along with PDGFRβ, EphA2 and AXL showed increase phosphorylation while HGFR, PDGFRα and EphB2 were dephosphorylated upon AZD1480 exposure (Fig. 2c, Additional file 4: Figure S3C). Among the phosphorylated proteins, HER3, HER4 and PDGFRβ showed significant fold changes (Additional file 4: Figure S3B). Recently PDGFRβ-mediated resistance to MEK1/2 inhibition through *MYC* suppression has been shown as an acute resistance mechanism in TNBC cells [37]. We therefore asked if PDGFRβ acts as a resistance mechanism upon JAK2 inhibition in our settings. To test this, we exposed both SUM159PT and MDA-MB-231 cell lines to different doses of JAK2 inhibitor, AZD1480 and found an increase in PDGFRβ protein levels along with a reduction in components of JAK2-MYC signaling (Fig. 2d). Likewise, we found that a single dose of AZD1480 increased PDGFRβ protein levels within 24 h of drug exposure (Additional file 4: Figure S3D). To exclude inhibitor specific effects, we challenged SUM159PT cells with varying concentrations of two additional JAK2 inhibitors, BSK-805 and Ruxolitinib and found a similar accumulation of PDGFRβ protein levels (Fig. 2e), suggesting this as a *bonafide* effect of JAK2 inhibition. MYC has been shown to occupy the promoter of PDGFRβ and suppresses its transcriptional activation [38]. Consistent with STAT3-mediated *MYC* expression [39], we noticed a significant accumulation of *PDGFRβ* transcript at higher concentration of JAK2 inhibition (5 μM) at which point *MYC* levels were significantly reduced (Pearson correlation coefficient, − 0.9842, *P* = 0.0158, Additional file 4: Figure S3E, F). However, PDGFRβ induction occurred prior to appreciable decline in MYC levels (Fig. 2d) and PDGFRβ transcript accumulation does not synergistically increase PDGFRβ protein levels in our settings. This suggests a *MYC*-independent effect of PDGFRβ accumulation upon JAK2 inhibition.

Consistent with the inhibitor experiments, the knockdown of JAK2 but to a lesser extent JAK1 markedly increased PDGFRβ protein levels in SUM159PT cells (Fig. 2f), suggesting that the loss of JAK2 signaling promotes PDGFRβ accumulation, similar to previous reports on MEK1/2, EGFR, HGFR and HER2 inhibition-mediated PDGFRβ accumulation [37, 40]. Furthermore, immunoblotting analysis showed that only two of the tested breast cancer cell lines (SUM159PT and Hs578T) exhibited marked accumulation of basal PDGFRβ levels (Fig. 2g) and the majority of the cell lines expressed very low to none PDGFRβ levels. Despite this observation, silencing of PDGFRβ using siRNA showed reduced cell viability in the majority of the TNBC lines but not in non-TNBC lines (Fig. 2h, Additional file 4: Figure S3G), implicating that TNBC cell lines are more likely dependent on PDGFRβ levels to a certain degree. To further determine the role of PDGFRβ in this resistance mechanism, we exposed PDGFRβ-expressing SUM159PT cells to various small molecule inhibitors or chemotherapeutic agents and found that inhibitors against MEK1/2, JAK2, FAK, and EGFR/HER2, along with doxorubicin induced PDGFRβ levels within 24 h of treatment (Fig. 2i).

To investigate this in a clinical context, we analyzed the relationship between *PDGFRβ* mRNA expression and the survival of breast cancer patients using KMPlotter datasets [19]. We found that high *PDGFRβ* expressing basal-like breast cancer patients (*n* = 421) exhibited significantly higher probabilities of relapse free survival than low *PDGFRβ* expressing patients (*n* = 197) [HR = 0.77 (0.69–1); *n* = 618; Logrank *P* = 0.049]. In comparison, with variable chemotherapy regimens, high *PDGFRβ* expressing patients (*n* = 101) exhibited significantly lower probability of survival than low *PDGFRβ* expressing patients (*n* = 300) [HR = 1.63 (1.13–2.35)] (Fig. 2j). We found similar results when *PDGFRβ* was analyzed across breast cancer patients irrespective of subtypes and expression of its ligand *PDGFB* in chemotherapy treated patients (Additional file 5: Figure S4A, B). As PDGFRβ expression was elevated upon treatment with a number of small molecule inhibitors and chemotherapeutic agent and plays a role in patient survival, we then asked if PDGFRβ inhibition reciprocally activates pro-survival signaling. Interestingly, we found that upon exposing SUM159PT cells to CP673451, a selective PDGFRβ inhibitor or the FDA-approved drug Imatinib, an ABL, C-kit and PDGFR inhibitor, components of pro-survival pathways were markedly activated within 24 h of treatment, suggesting interplay between PDGFRβ and pro-survival signaling (Fig. 2k). Taken together, our data suggested that PDGFRβ expression might act as a common resistance mechanism across small molecule inhibitors or chemotherapeutic agent in TNBC.

### JAK2-mediated PDGFRβ proteolysis in TNBC

**Fig. 3:**
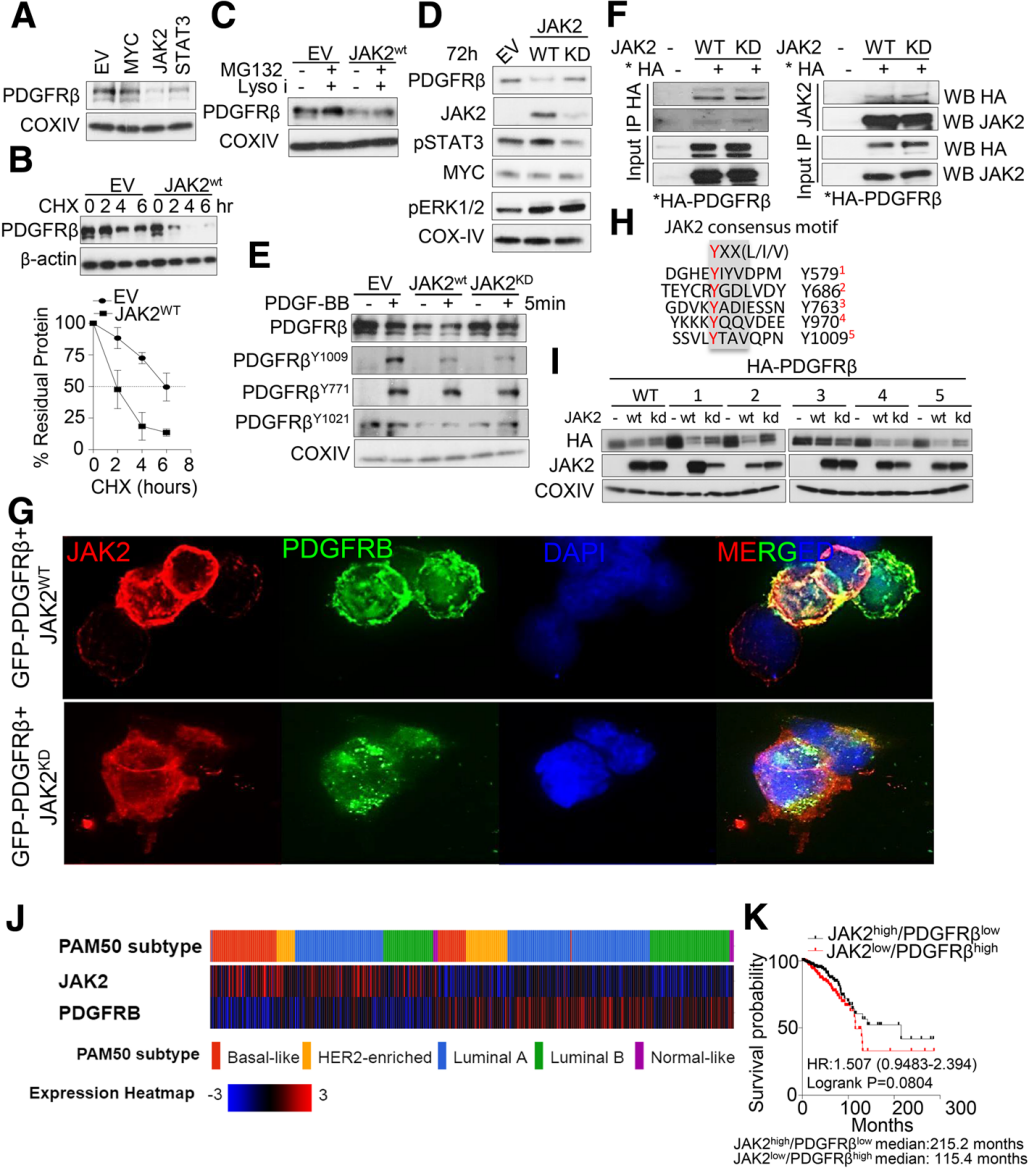
*JAK2 controls proteolysis of PDGFRβ.* **a** SUM159PT cells were reversed transfected with 1 μg of DNA of empty vector, MYC, mJAK2 or STAT3 using Lipofectamine 3000 for 24 h and PDGFRβ levels were determined by western blot. **b** Top: SUM159PT cells were reversed transfected with 1 μg of empty vector or mJAK2 for 24 h followed by 100 μg/ml cycloheximide (CHX) and cells were harvested at indicated time points. PDGFRβ and β-actin levels were determined by western blot. Bottom: Quantification of immunoblot images was performed using ImageJ software (NIH, Bethesda, MD, USA) and is presented in graphical form. The levels were normalized against β-actin, *n* = 3 with SEM. **c** SUM159PT cells were reversed transfected with 1 μg of empty vector or mJAK2 for 24 h followed by combination treatment with proteasomal (MG132) and lysosomal inhibitors (pepstatin and leupeptin) for 4 h. PDGFRβ levels were determined by western blot. **d** SUM159PT cells were reversed transfected with 1 μg of empty vector, mJAK2 wildtype or JAK2 kinase dead constructs for 24 h. PDGFRβ levels were determined by western blot. **e** SUM159PT cells were reversed transfected with 1 μg of empty vector, mJAK2 wildtype or JAK2 kinase dead constructs for 24 h followed by PDGF-BB (20 ng/ml) stimulation for 5 min. Phosphorylated PDGFRβ levels were determined by western blot. **f** HEK293T cells were reversed transfected with 1 μg of empty vector, mJAK2 wildtype or JAK2 kinase dead constructs for 24 h followed by combination treatment with proteasomal (MG132) and lysosomal inhibitors (pepstatin and leupeptin) for 4 h. Cell lysates were immunoprecipitated using either HA- or JAK2- specific antibodies and immunoblotted for indicated proteins. **g** Immunofluorescence analysis of PDGFRβ localization with JAK2. HEK293T cells were reversed transfected with wildtype or kinase dead mJAK2 constructs with GFP-tagged PDGFRβ expression constructs for 24 h, followed by combination treatment with proteasomal (MG132) and lysosomal inhibitors (pepstatin and leupeptin) for 4 h. Cells were fixed, permeabilized and stained with JAK2-specific antibody. **h** Sequence alignment of putative JAK2 consensus phosphorylation motif which recognizes YXX [L/I/V] in PDGFRβ. Possible Tyrosine sites are indicated in red font. **i** HEK293T cells were reverse transfected with wildtype and mutant PDGFRβ at indicated sites as shown in panel H in the absence and presence of mJAK2 wildtype or kinase dead construct. PDGFRβ levels were determined by western blot. **j** Heatmap analysis of correlation of *PDGFRβ* with *JAK2* levels in TCGA breast cancer samples. Patient samples were divided into PAM50 subtypes. Data derived from cbioportal (http://www.cbioportal.org/). **k** Breast cancer TCGA patient’s data (http://tumorsurvival.org) was divided into two subgroups based on *PDGFRβ* and *JAK2* expression and survival probability was plotted

In order to decipher the role of JAK2 inhibition-mediated PDGFRβ accumulation in breast cancer cells, we ectopically expressed plasmids encoding for transcription factors MYC [38] and STAT3 [39] that are known to regulate PDGFRβ levels as well as mJAK2, an upstream regulator of JAK/STAT signaling in SUM159PT cells for 24 or 72 h. Surprisingly we found that PDGFRβ levels were distinctly reduced upon JAK2 expression compared to MYC or STAT3 expression (Fig. 3a, Additional file 6: Figure S5A). Therefore, this data suggests that JAK2, which is a kinase, might regulate PDGFRβ steady state levels in breast cancer cells. To confirm this, we examined the role of JAK2-mediated PDGFRβ degradation by cycloheximide chase. The half-life of PDGFRβ in JAK2 transfected cells was markedly lower than the empty vector transfected cells (Fig. 3b). A number of reports have shown that receptor tyrosine kinases (RTKs) indeed undergo ligand-stimulated ubiquitination followed by trafficking through different intracellular compartments (i.e secretory pathway, plasma membrane, endosomes and lysosomes) for degradation [41, 42]. Likewise, Mori et al., showed that ligand-stimulated PDGFRβ undergoes ubiquitin-mediated proteasome degradation as inhibition of proteasome considerably inhibit ligand-stimulated PDGFRβ degradation [43]. However, we found that ectopic expression of JAK2 without ligand stimulation is sufficient to degrade PDGFRβ, suggesting this proteolysis is ligand-independent at basal condition (Fig. 3a). Next, we asked if addition of combined proteasomal (MG132) and lysosomal inhibitors (pepstatin and leupeptin) could prevent JAK2-mediated PDGFRβ degradation. Notably, both inhibitors increased PDGFRβ steady state levels at basal condition (Fig. 3c). To this end, we also found that JAK2 mediated PDGFRβ degradation is partly kinase-dependent as introduction of JAK2 kinase dead mutant prevented PDGFRβ degradation (Fig. 3d, e). Likewise, stimulation of PDGFRβ by its ligand PDGF-BB markedly reduced PDGFRβ levels within 5 min of receptor activation, as evident by the increased phosphorylation of PDGFRβ on Y1009 and Y771. Notably, ligand induced phosphorylation of PDGFRβ on Y1009 was impaired after expression of both wildtype and kinase-dead JAK2 (Fig. 3e). Next we investigated whether JAK2-mediated PDGFRβ degradation is a direct consequence of JAK2 kinase activation. Immunoprecipitation (IP) of HA tagged PDGFRβ in the presence of both proteasomal and lysosomal inhibitors showed direct interaction with both wildtype and kinase-dead JAK2 and reciprocally PDGFRβ was detected in JAK2 immunoprecipitate from HEK293T cells (Fig. 3f). We used PDGFRβ-null HEK293T cells in this case for better transfection efficiency. Likewise, immunofluorescence analysis of ectopically expressed GFP-tagged PDGFRβ showed a remarkable co-localization with wildtype JAK2 but not with kinase dead JAK2 (Fig. 3g), suggesting a direct regulation of PDGFRβ steady state levels as well as localization at the membrane by JAK2 kinase.

To this end, we asked if tyrosine kinase JAK2, which recognizes YXX[L/I/V] motif for phosphorylation of substrate, may be responsible for PDGFRβ phosphorylation and consequent degradation. We found that JAK2 recognizes five consensus sites within PDGFRβ including Y1009 phosphorylation (Fig. 3h). To test whether JAK2-mediated phosphorylation on PDGFRβ promotes its proteolysis, we substituted tyrosine residues to phenylalanine by site directed mutagenesis in all JAK2 consensus sites within PDGFRβ. Expression of Y763F mutant compared to other mutants seems to partly prevent JAK2-dependent PDGFRβ proteolysis (Fig. 3i). Finally, we found an inverse correlation between *JAK2* and *PDGFRβ* mRNA levels in both breast cancer TCGA and METABRIC data (Fig. 3j and Additional file 6: Figure S5B). We also found similar trends between *JAK2* and *PDGFRβ* expression levels using pan-TCGA datasets (Additional file 6: Figure S5B). Moreover, although not statistically significant, we found a pattern of poor survival in TCGA breast cancer patients expressing high *PDGFRβ* with low *JAK2* levels compared to patients expressing low *PDGFRβ* with high *JAK2* levels (HR:1.507, logrank: 0.00804) (Fig. 3k). Notably, these patients survive much longer than patients expressing high *PDGFRβ* with low *JAK2* (215.2 months vs. 115.4 months) (Fig. 3k). This data cumulatively suggests a direct, yet novel regulation of PDGFRβ levels by JAK2 in breast and other cancers.

### PDGFRβ inhibition circumvented resistance to combined MEK1/2-JAK2 inhibition

**Fig. 4:**
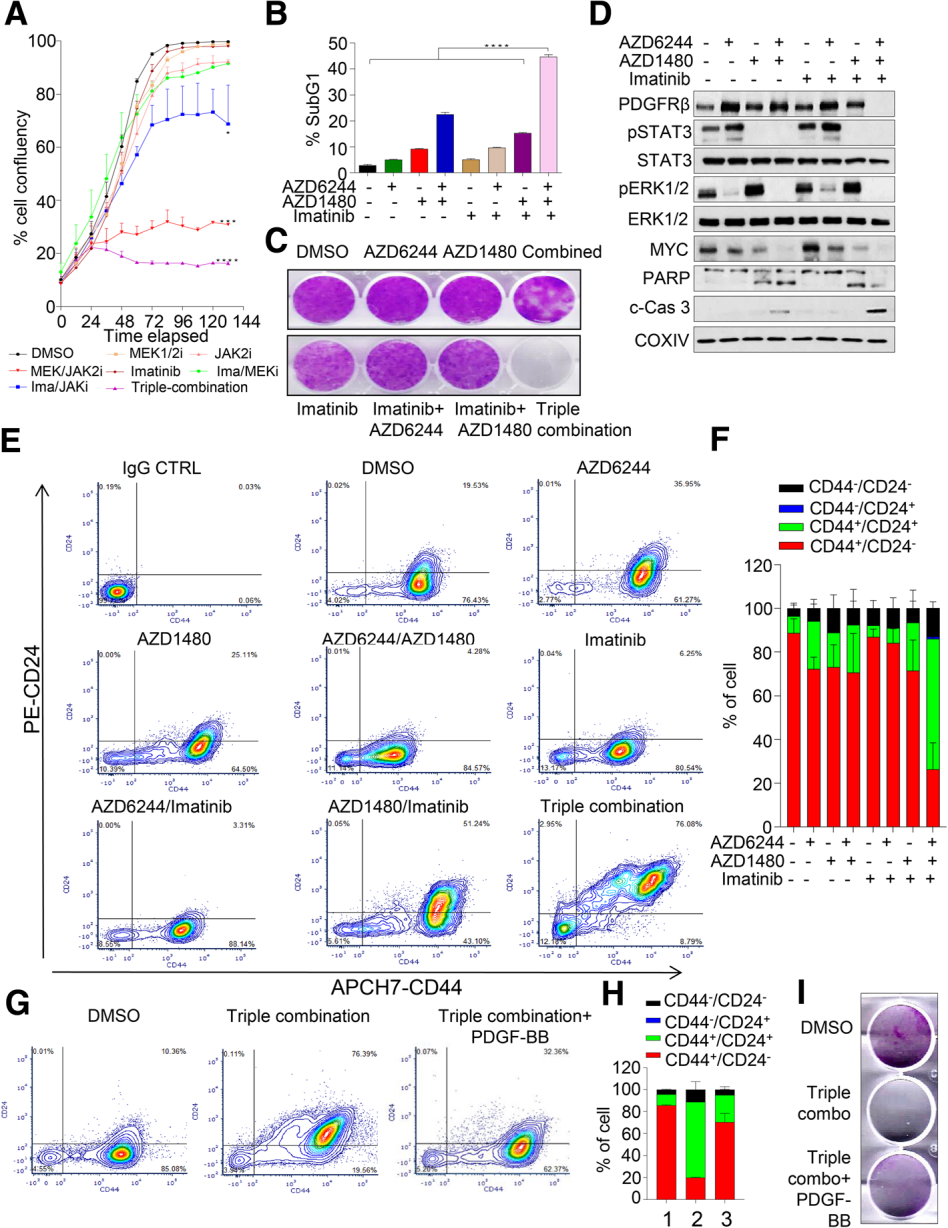
*PDGFRβ inhibition enhances efficacy of MEK1/2-JAK2 inhibition in TNBC cells.* **a** Effect of single, double and triple combination treatment with AZD6244 (1 μM), AZD1480 (2.5 μM) and Imatinib (5.0 μM) inhibitors on cell proliferation in SUM159PT cells assessed using the IncuCyte ZOOM® live-cell imager (phase-only processing module). The percentage of cell confluence was determined using an IncuCyte mask analyser, *n* = 2 with SEM (****p < 0.0001). **b** Percentage of sub-G1 population identified using propidium iodide staining and quantified by FACS following indicated concentration of inhibitors as in panel A after 72 h, *n* = 2 with SEM (****p < 0.0001). **c** Representative images of colony-forming capacity at 14 days determined using crystal violet staining in SUM159PT cells treated indicated concentration of inhibitors as in panel A. **d** SUM159PT cells treated with indicated concentration of inhibitors as in panel **a** for 72 h and western blot was performed to determine the levels of indicated proteins. **e**, **g** Representative images of contour plot of stem cell-like cell population following single, double and triple combination for 72 h with respective drug concentrations as indicated in panel **a**. Percentage of CD24 and CD44 were determined using conjugated antibodies as indicated in x- and y-axes. **f**, **h** Quantification of each subpopulation of cells as in panel **e** and **g**, *n* = 2–3 with SEM. **i** Representative images of colony-forming capacity at 14 days determined using crystal violet staining in SUM159PT cells treated indicated concentration of inhibitors as indicated in panel **a** and/or stimulated with 10 ng/ml of PDGF-BB ligand

Next, we sought to determine the combined efficacy of triple combination inhibition in representative TNBC lines. Exposure of SUM159PT, MDA-MB-231 and Hs578T cells to 5 μM of Imatinib significantly enhanced the efficacy of MEK1/2-JAK2 inhibition in blocking proliferative capacity (Fig. 4a, Additional file 7: Figure S6). However, exposure of Imatinib alone had no significant anti-proliferative effect. Moreover, the sub-G1 population of this triple combination treatment was significantly higher than single and dual combination treatments (Fig. 4b, Additional file 7: Figure S6A) with complete loss of colony forming capacity in the triple-combination treated group (Fig. 4c, Additional file 7: Figure S6C). Notably, this triple-combination therapy also markedly induced cleaved PARP and Caspase 3 in contrast to dual or single treatments (Fig. 4d, Additional file 7: Figure S6B).

Basal-like breast cancer is a heterogeneous disease and cancer stem-like cells play a pivotal role in resistance against small molecule inhibitors in this disease setting [44]. To determine whether PDGFRβ inhibition eliminates stem cell-like cell subpopulation, we exposed SUM159PT cells with single, dual and triple combination inhibitors for 72 h to determine the percentage of CD24^−^/CD44^+^ stem like sub-population. Notably, high PDGFRβ-expressing SUM195T cells were enriched with a CD24^−^/CD44^+^ sub-population and this subpopulation was significantly reduced with concomitant enrichment of a more differentiated CD24^+^/CD44^+^ subpopulation upon triple combination therapy (Fig. 4e, f). For example, in basal conditions, ~ 19% of cells were CD24^+^/CD44^+^ which were significantly reduced upon JAK2-MEK1/2 inhibition (~ 4%). Almost three quarter of cells exhibited CD24^+^/CD44^+^ double positive staining upon triple combination treatment and to a lesser extent (~ 50%) in a group treated with combined PDGFRβ and JAK2 inhibitors. Similar data was also observed in high PDGFRβ-expressing HS578T cell lines (Additional file 7: Figure S6D). Our data thus suggests that even though dual combination of MEK-JAK inhibition showed some degree of growth suppression (Fig. 4a), it does not impact on cancer stem cell contents; however, additional suppression of PDGFRβ is able to dramatically reduce this subpopulation. To validate the contribution of PDGFRβ in acquired resistance in this setting, we pre-treated breast cancer cells with triple combination therapy for 72 h, washed out the inhibitors and further stimulated cells with PDGF-BB ligands for a further 48 h. We found that upon PDGF-BB stimulation, the triple-inhibitor treated CD24^+^/CD44^+^ double positive cells significantly reverted back to CD24^−^/CD44^+^ stem-like cell populations, however cells without PDGF-BB stimulation died in culture (Fig. 4g, h, Additional file 7: Figure S6E). Moreover, PDGF-BB stimulation also rescued apoptotic phenotypes with an increase in cell viability and colony numbers (Fig. 4i, Additional file 7: Figure S6E, F). Overall, this data suggests a strong contribution of PDGFRβ in mediating resistance upon small molecule inhibitors probably through modulating stem cell-like cell subpopulations and apoptosis in TNBC cells.

### Intratumoral CD8^+^ T cells enhanced the efficacy of triple combined inhibition in-vivo

**Fig. 5:**
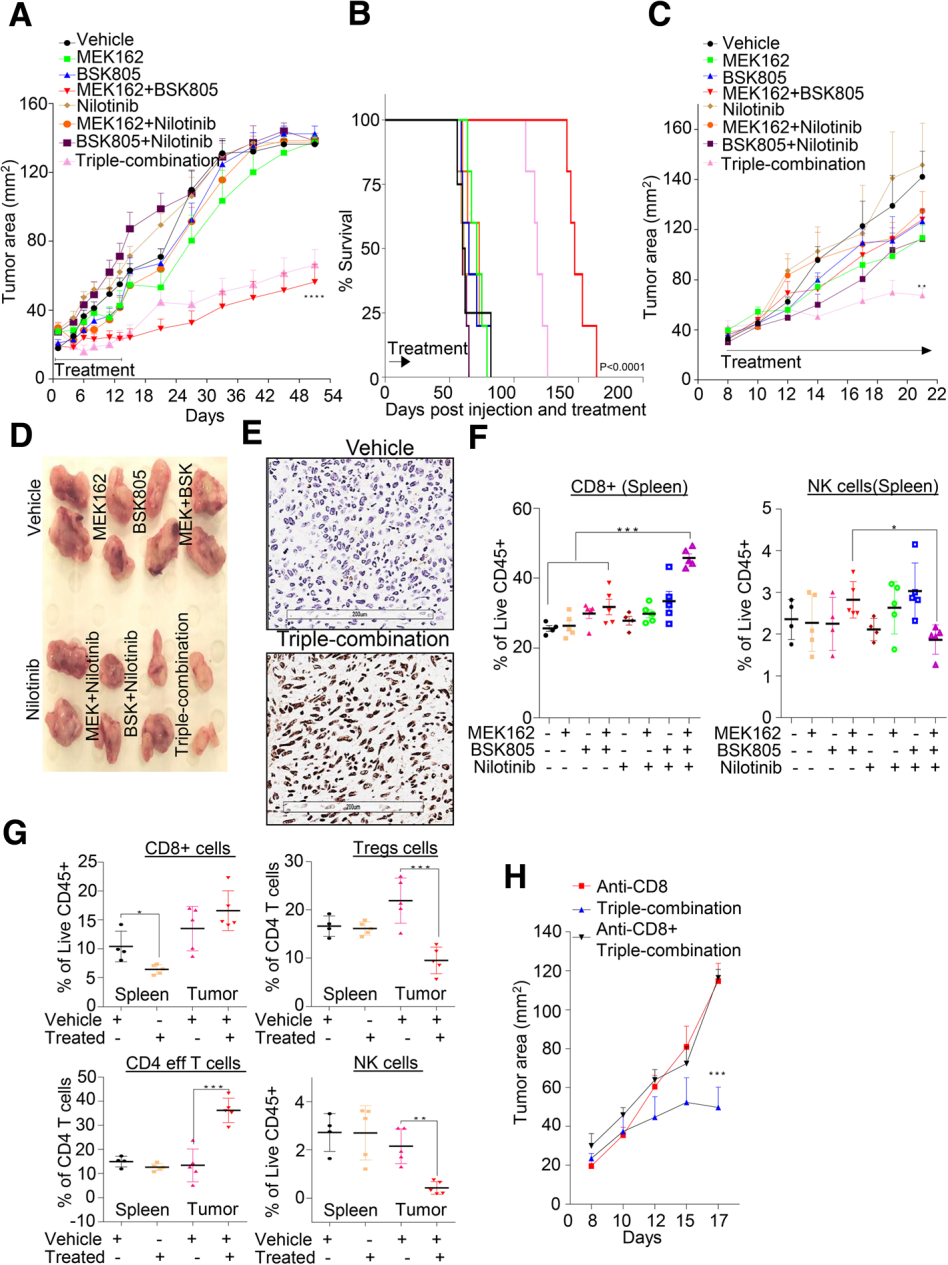
*Co-blockade of PDGFRβ with MEK-JAK inhibition enhances tumor cell killing* via *intratumoral T-cells* in vivo*.* **a** 6-week-old female BALB/c nude mice cohorts were injected in the 4th inguinal mammary fat pad with the MDA-MB-231 cells line. Mice were treated with vehicle, MEK162 (5 mg/kg), NVP-BSK805 (50 mg/kg), Nilotinib (37.5 mg/kg) individually or in combination for 14 days. Tumor size (area, mm^2^) was measured using a digital calliper and mean tumor size of each cohort is presented. Graph represents the mean tumor area ± SEM from six mice/group (****P ≤ 0.0001). **b** Survival of mice of panel **a** was monitored over the indicated period of time and the statistical significance of data was analyzed by log-rank test (*P* < 0.0001); *n* = 6 mice/group. **c** Similarly to panel **a**, syngeneic mammary carcinoma cell line 4 T1.2 model was established using 6-week-old female BALB/c mice and tested with indicated inhibitors. Graph represents the mean tumor area ± SEM from six mice/group (****P ≤ 0.01). **d** Representative images of gross morphology of excised tumor are shown for panel **c**. **e** Representative images of ApopTag staining in tumors treated with vehicle and triple-combination therapy. **f**, **g** Percentage of viable immune cells infiltrates gated using indicated antibodies as shown in Additional file 8: Figure S7F in both spleens and tumor tissues isolated from indicated treatment groups. Graph represents each cell population from six mice/group± SEM (*P ≤ 0.05, **P ≤ 0.01, ***P ≤ 0.001). **h** Syngeneic 4 T1.2 cancer model as in panel **c** was established and treated singly or in combination with anti-CD8 or triple-combination. Tumor size (area, mm^2^) was measured using a digital caliper and mean tumor size of each cohort is presented. Graph represents the mean tumor area ± SEM from six mice/group (***P ≤ 0.001)

To determine whether PDGFRβ signaling blockade enhances the growth-inhibitory effect of combined MEK1/2-JAK2 inhibition (AZD6244/AZD1480) in basal-like breast cancer cells in vivo, we first tested the combination therapy using a human cell line, MDA-MB-231 xenografts in immunocompromised nude mice. The combined MEK1/2-JAK2 inhibition (evaluated through Novartis inhibitors of MEK162 + NVP-BSK805) significantly reduced the tumor growth and enhanced the survival of tumor bearing mice, hence survival (Fig. 5a, b); however, the tumor started to resume while on treatment, suggesting a resistance phenotype. Unexpectedly, the addition of PDGFRβ inhibitor Nilotinib did not provide any additional suppression of tumor growth either alone or when combined with MEK1/2-JAK2 inhibitors in double or triple combination therapy. Though we did observe a significant regression in tumor growth until day six; however, the tumor growth seemed to resume while on treatment and upon therapy withdrawal (Fig. 5a).

Recent report suggests that JAK2 inhibition increases metastasis burden by suppressing the function of NK cells in breast cancer [45] and off-targets effects of Nilotinib on various immune cells may contribute towards its anti-tumor efficacy [46]. Therefore, we hypothesize that immune components have a role in modulating the efficacy of MEK1/2-JAK2/PDGFRβ inhibition in our settings. For this, we first tested the combination therapy using fully immunocompetent murine preclinical syngeneic model.  4T1.2 is a murine basal-like breast cancer cell line that faithfully recapitulates human basal-like breast tumor phenotypes after orthotopic mammary fat pad implantation in immunocompetent Balb/c mice [47]. Firstly, we validated the effect of various inhibitors on  4T1.2 cells in vitro and found that triple combination therapy significantly killed most of the cells, as evident by the accumulation of sub-G1 population, cleaved Caspase 3 and complete reduction of colony forming capacity (Additional file 8: Figure S7A-C). Next, we injected  4T1.2 cells into Balb/c mice and treated them with similar doses as to human MDA-MB-231 xenograft model. The  4T1.2 syngeneic tumors significantly responded to MEK1/2 and JAK2 inhibitors, but not to PDGFRβ inhibitor Nilotinib, when used individually. Consistently, these tumors did not respond to various dual combination treatments (Fig. 5c). As expected the addition of PDGFRβ inhibitor Nilotinib with MEK1/2-JAK2 inhibition in triple combination therapy significantly suppressed the tumor growth compared to the MEK1/2-JAK2 double combination therapy due to induction of apoptosis, as evident by Apoptag staining (Fig. 5c-e). To understand the role of immune system in triple combination therapy, we collected spleens of the treated cohorts at end of the experiment (day 22) and various immune cell markers were examined (Fig. 5f, Additional file 8: Figure S7E, F, for gating strategy see Additional file 8: Figure S7D). Notably, we found a significantly higher proportion of CD8^+^ cytotoxic T cells in the triple-combination treated group compared to other treated groups in the spleen samples (Fig. 5f). We also found that the proportion of NK cells was relatively reduced in the triple combination treated cohort (Fig. 5f). Interestingly, dual inhibition of MEK/1/2-JAK2 did increase the proportion of NK cells and this was significantly reduced upon PDGFRβ inhibition (Fig. 5f). In line with this, Barrow et al. have recently shown the role of PDGF signaling in NK cell mediated tumor cell growth arrest via secretion of interferon gamma and tumor necrosis factor alpha [48].

Initially, due to marked differences in the tumor sizes of the different treatment groups (Fig. 5d), we did not determine the percentage of immune cell infiltrations in them. To measure various immune cell proportions in both tumors and spleens, we repeated this experiment and collected spleen and tumor samples at four days after treatment when tumor mass was about similar size across the treatment groups. As shown in Fig. 5g and Additional file 8: Figure S7F, various immune cell markers were examined. Although not statistically significant but we did find higher frequency of CD8^+^ T cells in tumor tissues within four days of triple combination treatment with a significant decrease in this proportion in corresponding spleens (Fig. 5g). Regulatory T cells (Tregs) are a population of immune cells that limit the anti-tumor function of CD8^+^ T cells and contribute towards increased tumor growth. We noticed a significant reduction in the frequency of Tregs and NK cells in tumors but not in spleens after triple combination therapy (Fig. 5g). To determine if CD8^+^ T cells were primarily responsible for the observed anti-tumor efficacy in triple combination therapy group, we depleted CD8^+^ T cells as previously reported [49]. Depletion of CD8^+^ T cells significantly reduced the anti-tumor efficacy of triple combination treatment (Fig. 5h) suggesting that CD8^+^ cytotoxic T cells are critical for anti-tumor efficacy of MEK1/2-JAK2/PDGFRβ inhibition in triple combination therapy. Cumulatively, our data suggests that PDGFRβ inhibition can evade the resistance to a combined MEK1/2-JAK2 inhibition and can enhance tumor suppression in immunocompetent mice. Taken together our data suggest that intratumoral CD8^+^ T cells largely contribute to this observed anti-tumor efficacy of triple combination in vivo.

## Discussion

Drug resistance still poses a significant clinical obstacle in achieving durable disease control as tumor cells often overcome target inhibition by activating parallel or downstream pathways that allow autonomous growth in the absence of direct signaling. Mechanisms of drug resistance have been linked to a subway map: blocking a commuter line will have consequences throughout the network as passengers try to find alternative routes to their destinations. Since oncogenes and other ‘disease-associated’ genes are generally part of larger networks within a cell, targeting multiple pathways provide a good strategy in combating recurrent and relapsed diseases. Here, we demonstrated a hypothesis driven combination therapy in TNBC to counteract resistance mechanisms by integrating studies from cell lines, xenografts and publically available clinical datasets and presents evidence of PDGFRβ-mediated resistance in TNBC cells upon JAK2 inhibition.

Kong et al. recently reported a combination inhibition of JAK and MEK in controlling juvenile myelomonocytic leukemia (JMML) and the myeloproliferative variant of chronic myelomonocytic leukemia (MP-CMML) that prevented T-ALL development in *NrasG12D/G12D* mice [50]. Moreover, another recent report shows that blocking KRAS-dependent ERK1/2 signaling in colorectal cancers activates JAK/STAT3 signaling [35]. We asked if similar activation occurs in TNBC cells following MEK1/2 inhibition. Recent preclinical study indicates that inhibition of MEK in TNBC is compensated by upregulation of other survival signaling pathways [37]. Similarly to the previous study, we found that upon exposure to MEK1/2 inhibitors, TNBC cells exhibited an activation of JAK-STAT3 signaling, suggesting different cancers behave similarly to target inhibition. However, unlike in colorectal cancer, we found a substantial number of resistant colonies arise following combination of MEK1/2-JAK2 inhibition in TNBC cell lines, suggesting a complexity in response in which TNBC cells are able to bypass combined inhibition of these two pathways to survive. We found that the EGFR family of proteins (HER3 and HER4) as well as PDGFRβ were markedly activated following JAK2 inhibition. Since there is accumulating interest in understanding PDGFRβ-mediated resistance mechanisms in various cancers, in this study we sort to investigate its role in resistance to JAK2 inhibition in breast cancer.

PDGFRβ belongs to the type III family of tyrosine kinase receptors which become dysregulated in various pathologies including cancer [51]. The PDGFRβ receptor consists of five immunoglobulin (Ig)-like extracellular domain, a single transmembrane segment, a juxtamembrane segment, a protein-tyrosine kinase domain and a carboxyl-terminal tail. PDGF ligands binding to its receptor result in the activation of its intrinsic tyrosine kinase activity followed by trans-autophosphorylation. This creates docking sites for the SH2-domain containing molecules including tyrosine kinases of the SRC family, the SHP-2 tyrosine phosphatase, phospholipase C-γ (PLC- γ) and the GTPase activating protein (GAP) for Ras to regulate various signaling pathways including PI3K and ERK-MAPK that are involved in proliferation and survival. Likewise, it also binds to the STAT family of transcription factors [52, 53].

Historically, *PDGFRβ* transcript expression is well known to be activated by many stimuli to promote targeted therapy-mediated resistance in various cancers [37, 40, 54–56]. One pathway implicated in *PDGFRβ r*egulation is MYC, a transcriptional repressor of *PDGFRβ*, hence blocking ERK1/2-dependent MYC signaling induces expression and activation of *PDGFRβ* in breast cancer [37]. Additionally, mutant EGFRvIII has been shown to suppress *PDGFRβ* expression via mTORC1- and ERK-dependent mechanisms and blocking such pathways de-repressed PDGFRβ signaling for growth and survival in glioblastomas [55]. Moreover, PDGFRβ has been shown to mediate Vemurafenib resistance through transcriptional upregulation in melanoma [40]. Here we provide evidence for the first time that PDGFRβ expression is proteolytically controlled by JAK2 kinase. Consistent with this, blocking JAK2 pharmacologically or genetically results in increased protein stability of PDGFRβ in TNBC. In alliance with its role in chemotherapy resistance [17], we also found an increase in PDGFRβ expression upon doxorubicin treatment in breast cancer cells. Notably, chemotherapy treated TNBC patients expressing high levels of *PDGFR*β or its ligand *PDGFB* exhibited poor relapse free survival when compared to *PDGFRβ* low expressing patients.

We found that the addition of a PDGFRβ inhibitor enhances the efficacy of MEK1/2-JAK2 inhibitors in killing TNBC cells through apoptosis and completely eliminated resistance colonies seen after combined MEK1/2-JAK2 inhibition. Cancer stem cell-like cell population play pivotal roles in tumorigenesis by facilitating heterogeneity, therapeutic resistance and metastasis [57]. Eradication of this subpopulation of cells in cancer treatment is paramount for a better cancer treatment efficacy, but to date little success has been seen in targeting sub-population of stem cell-like cells due to their plasticity. We found that upon triple-combined therapy, stem cell-like subpopulation was significantly reduced. Likewise, stimulation of triple-combined treated cells with PDGF-BB significantly restored their number and rescued apoptosis, providing rationale to include PDGFRβ inhibitor in combination with targeted small molecule inhibitors for therapeutic evaluation.

We also found that the triple-combined therapy significantly reduced syngeneic tumors growth due to immunogenic tumor cell death. Importantly, this effect was primarily mediated by intratumoral CD8^+^ cells as well as a reduction in Tregs cells within the tumor tissues. Likewise, anti-CD8 blocking antibody completely impaired the triple combination efficacy. Consistent with this, the presence of Tumor-infiltrating lymphocytes (TILs) including CD8^+^ T cells has been shown to be associated with longer survival in ER-negative tumors, particularly in TNBC [58–60]. Moreover, infiltration of TILs after neoadjuvant chemotherapy exhibited prolonged survival in TNBC patients [61, 62]. Notably, we failed to achieve durable response upon triple combination therapy in the human MDA-MB-231 xenograft model, further suggesting the observed efficacy is probably mediated by infiltrating T cells. In contrast, we did observe an enhanced efficacy with dual MEK1/2-JAK2 dual inhibitors combination treatment, suggesting that blocking JAK/STAT autocrine signaling in MEK1/2 inhibited cells did provide an advantage in this immunocompromised model. However, it is still underdetermined if immune cells have a role in triple-combined efficacy in humanized patient-derived xenograft models. Moreover, it also remains to be seen if the addition of immune checkpoint blockade inhibitors (i.e. anti- PD-1/PD-L1 or -CTLA-4/B7–1/B7–2) would enhance the response seen with triple-combination therapy.

Currently, we do not understand how PDGFRβ blockade with dual JAK2 and MEK1/2 inhibition augmented the infiltration of the CD8^+^ T cells in our model. PDGFR blockade either by imatinib or nilotinib has been shown to have an inhibitory effect on T cells proliferation and survival [63, 64]; however we failed to see such reduction in percentage of CD8^+^ T cells in spleen of 4 T1.2 nilotinib treated tumours. In addition, few recent reports suggest that various JAK inhibitors reduce T-reg populations while augment antitumor activity of CD8^+^ T cells, macrophages and NK cells [65, 66], and MEK1/2 inhibition promotes T cell and anti-tumour activity in combination with PD-L1 checkpoint blockade [67]. This suggests rather a complex contribution of different inhibitors towards CD8^+^ T cells infiltration in our model. Further studies are therefore necessary to understand the role of tumour microenvironment in enhancing the efficacy of triple combination inhibition.

## Conclusion

**Fig. 6:**
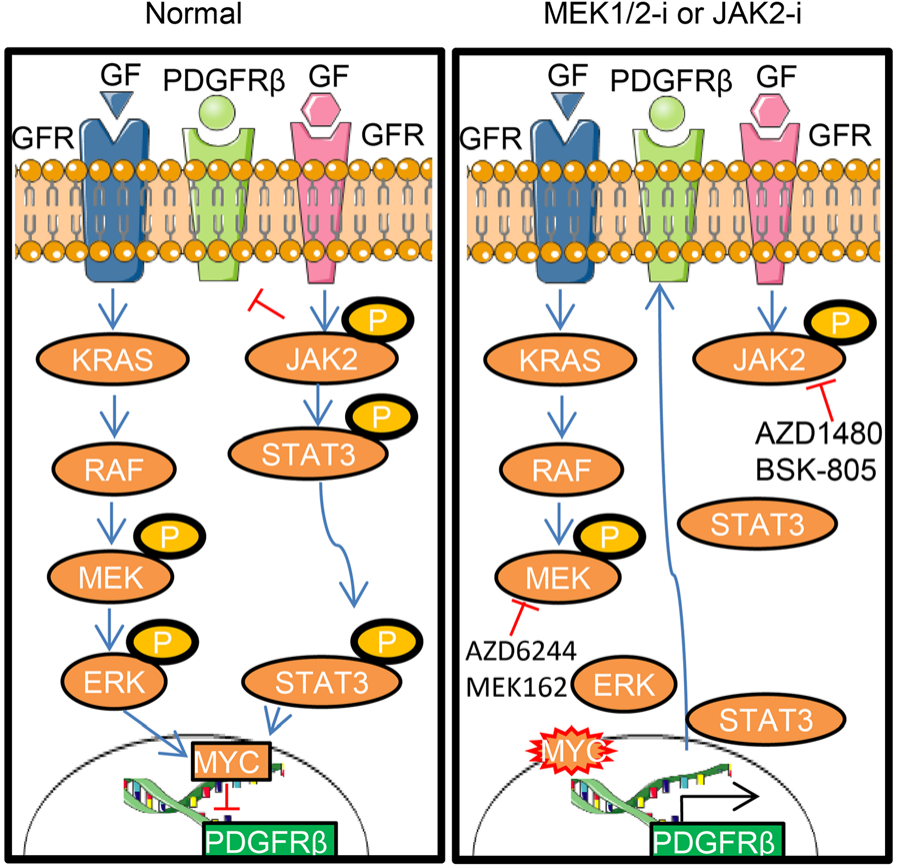
*Schematic model of PDGFRβ switch upon MEK1/2 or JAK2 inhibition in TNBC cells.* Under normal physiological condition, PDGFRβ expression is transcriptionally controlled by *MYC* through MAPK signaling and its protein level is controlled through JAK2 mediated proteolysis (left panel). Upon targeted inhibition of these signaling pathways (i.e. AZD6244, MEK162, BSK805 or AZD1480) results in accumulation of PDGFRβ, driving resistance in breast cancer (right panel), GF: Growth factor; GFR: Growth factor receptor

According to our model (as illustrated in Fig. 6), PDGFRβ levels are controlled by both MAPK and JAK-STAT signaling as two distinct and shared mechanisms. Under physiological state, *MYC* suppresses *PDGFRβ* transcriptionally [38], and therefore blocking MYC through MEK1/2 inhibitors enhances PDGFRβ transcripts. We also found that at higher concentrations of JAK2 inhibitor, *PDGFRβ* transcription was elevated, most likely through the suppression of *MYC*. Consistent with this finding, STAT3 has been shown to regulate MYC activity and vice versa [39, 68]. Moreover, in this current study we demonstrated that JAK2 also regulates the stability of PDGFRβ, partly in a kinase-dependent manner by targeting it for degradation. To our knowledge, this is the first report showing JAK2 mediated proteolysis of PDGFRβ steady state levels in breast cancer. We observed JAK2-dependent phosphorylation of PDGFRβ at Tyrosine 763 as mutant PDGFRβ (Y to E763) was partly refractory to degradation by JAK2. PDGFRβ plays a pivotal role in resistance mechanisms against various small molecules inhibitors. However, PDGFRβ inhibition alone has shown little effect on tumor growth. This suggests an autocrine signaling mediated by PDGFRβ that facilitates the growth of resistant colonies. Therefore, the addition of a PDGFRβ inhibitor to existing targeted agents could potentially aid in obtaining a durable response in the clinic and should be considered in future clinical trial evaluations of combination-targeted therapy across multiple cancers.

## Additional files

Additional file 1: **Table S1.** List of small interfering RNA (siRNA), primers for mutagenesis, primers for RT-qPCR and antibodies used in this study. (XLSX 15 kb)

Additional file 2: **Figure S1:** (A) Dysregulation score was calculated according to Pathifier [32] for MAPK and JAK/STAT signaling in breast cancer using TCGA dataset. (B, C) A combined panel of selected breast cancer and near-normal cell lines was reverse-transfected with 10 nM pooled *JAK1, JAK2, STAT3* or *KRAS* siRNAs and cell viability determined after 6 days. Cell viability relative to its own respective control transfected with scramble siRNA was calculated, *n* = 2–3 (*p < 0.05, *p* < 0.01). Individual cell lines are shown in panel C. (JPG 2069 kb)

Additional file 3: **Figure S2:** (A) Hs578T cells were exposed to different concentrations of MEK1/2 inhibitor (AZD6244) alone or in combination with JAK2 inhibitor (2.5 μM) and cell viability was determined after 6 days using MTS assays. The dose-response curve was generated by calculating cell viability relative to untreated control and plotted against drug concentration, *n* = 3 with SEM (***p < 0.001). (B,C, D) Immunoblots analysis in indicated panel of breast cancer cell lines treated with single and combination treatments after 48 h and indicated proteins were determined. (JPG 2427 kb)

Additional file 4: **Figure S3:** (A,B) Prediction of Kaplan Meier survival analysis in TCGA patients using data derived from cell line treated with Ruxolinitib and computed based on machine-learning (ML) model. See methodology for further details. (C) Relative fold change of phospho-RTK levels derived from Fig. 2c. Quantification of protein-band intensities by densitometric analysis was performed using NIH ImageJ software (NIH, Bethesda, MD). Internal controls of within the array were used to normalize the phosphorylated protein levels. Fold changes are indicated in the graph. (D) SUM159PT cells were treated 2.5 μM AZD1480 as indicated time points and indicated proteins were determined by western blot. (E) Relative fold change of *PDGFRβ* and *MYC* mRNA expression upon SUM159PT cells treated with different concentration of AZD1480, *n* = 3 with SEM (**P ≤ 0.01, ***P ≤ 0.001). (F) Pearson correlation coefficient was calculated between *PDGFRB* and *MYC* expression using data derived from panel E. (G) A panel of selected breast cancer and near-normal cell lines was reverse-transfected with 10 nM pooled *PDGFRβ* siRNAs and cell viability determined after 6 days. Cell viability relative to its own respective control transfected with scramble siRNA was calculated, *n* = 2–3. (JPG 2271 kb)

Additional file 5: **Figure S4:** (A, B) Kaplan-Meier survival analysis of the relationship between *PDGFRβ* or *PDGFB* mRNA expression and clinical outcomes in breast cancer patients treated with and without chemotherapy using the KMplotter dataset (http://kmplot.com/). *PDGFRβ* expression stratified on relapse free survival. (JPG 2951 kb)

Additional file 6: **Figure S5:** (A) SUM159PT cells were reversed transfected with 1 μg of DNA of empty vector, mJAK2 or STAT3 using Lipofectamine 3000 for 72 h and PDGFRβ levels were determined by western blot. (B) Heatmap analysis of correlation of *PDGFRβ* with *JAK2* levels in pan-TCGA cancer samples. Patient samples were divided into low and high expression. Data derived from cbioportal (http://www.cbioportal.org/). (JPG 1842 kb)

Additional file 7: **Figure S6:** (A) Percentage of sub-G1 population identified using propidium iodide staining and quantified by FACS upon MDA-MB-231 and HS578T cells treated AZD6244 (1 μM), AZD1480 (2.5 μM) and Imatinib (5.0 μM) inhibitors after 72 h, *n* = 2 with SEM (****p < 0.0001). (B) MDA-MB-231 and HS578T cells treated with indicated concentration of inhibitors as in panel A for 72 h and western blot was performed on to determined levels of indicated proteins. (C) Representative images of colony-forming capacity at 14 days determined using crystal violet staining in MDA-MB-231 and HS578T cells treated indicated concentration of inhibitors as in panel A. (D, E) Quantification of percentage of CD24 and CD44 in Hs578T cells were determined as indicated in Fig. 4e and f, *n* = 2–3 with SEM. 1: DMSO; 2: Triple-combination; 3: Triple combination with 10 ng/ml of PDGF-BB. (F) Representative Images of colony-forming capacity at 14 days determined using crystal violet staining in SUM159PT cells treated indicated concentration of inhibitors as indicated in panel E and/or stimulated with 10 ng/ml of PDGF-BB ligand. (JPG 3786 kb)

Additional file 8: **Figure S7:** (A) Percentage of sub-G1 population identified using propidium iodide staining and quantified by FACS upon 4 T1.2 cells treated AZD6244 (1 μM), AZD1480 (2.5 μM) and Imatinib (5.0 μM) inhibitors after 72 h, *n* = 2 with SEM (****p < 0.0001). (B) 4 T1.2 cells treated with indicated concentration of inhibitors as in panel A for 72 h and western blot was performed on to determined levels of indicated proteins. (C) Representative images of colony-forming capacity at 14 days determined using crystal violet staining in 4 T1.2 cells treated indicated concentration of inhibitors as in panel A. (D) Gating strategies to identify subpopulation of immune cells using specific antibodies as indicated within the Fig. (E, F) Percentage of viable immune cells infiltrates gated using indicated antibodies in both spleens and tumor tissues isolated from indicated treatment groups. Graph represents each cell population from six mice/group± SEM (*P ≤ 0.05, **P ≤ 0.01, ***P ≤ 0.001, ****P ≤ 0.0001). (JPG 4558 kb)

## Abbreviations

CD8: Cluster of differentiation 8
JAK2: Janus kinase 2
PDGFRβ: Platelet-derived growth factor receptor beta
RTK: Receptor tyrosine kinase
RT-PCR: Real time- polymerase chain reaction
TNBC: Triple-negative breast cancer
