# Supplementary figures and images for: Blockade of PDGFRβ circumvents resistance to MEK-JAK inhibition via intratumoral CD8+ T-cells infiltration in triple-negative breast cancer
Source: J Exp Clin Cancer Res. 2019 Feb 18;38:85. doi: 10.1186/s13046-019-1075-5 (PMC6379987; doi:10.1186/s13046-019-1075-5)

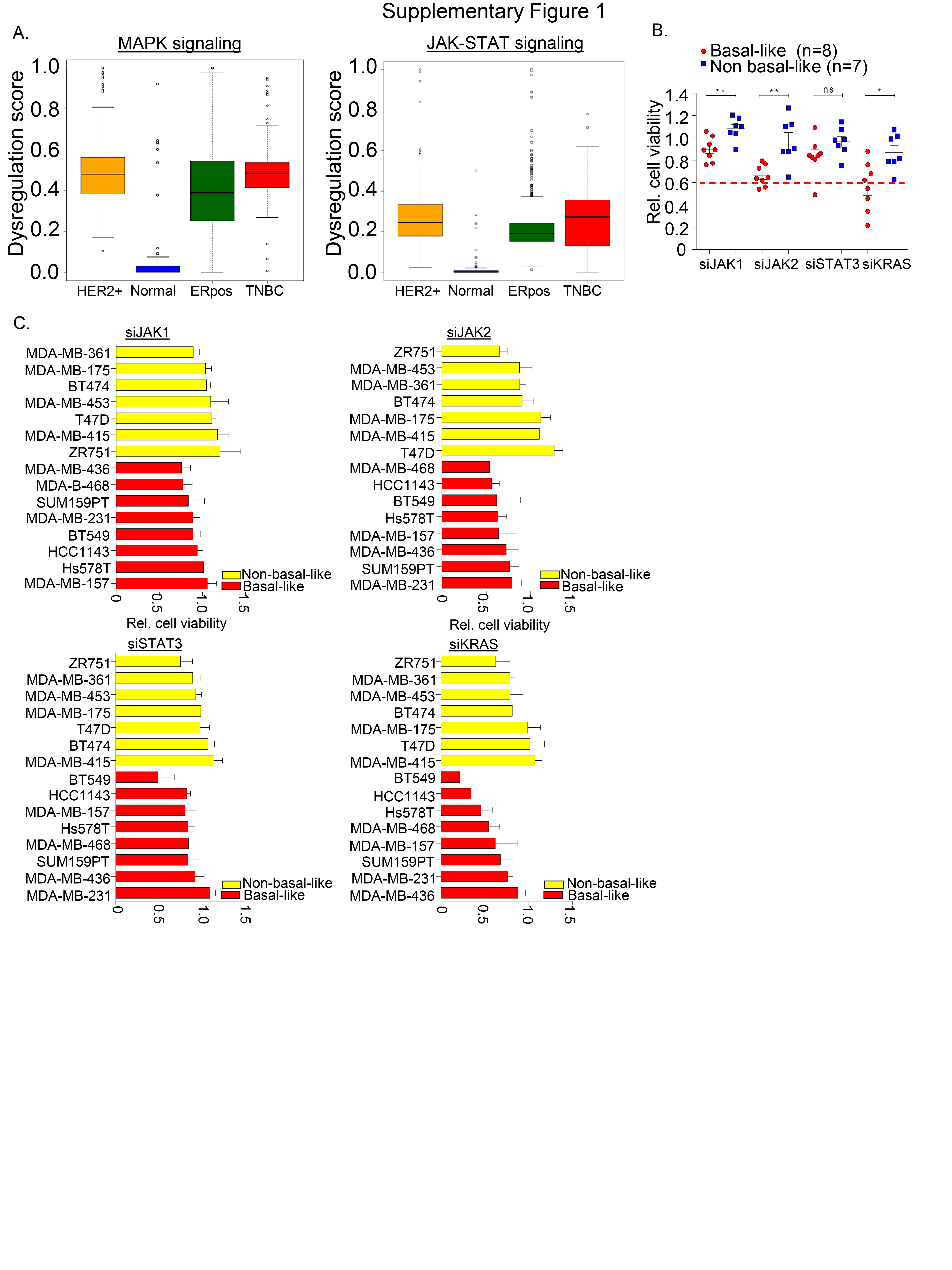

Supplement: Supplementary file 2 — Figure S1: (A) Dysregulation score was calculated according to Pathifier [32] for MAPK and JAK/STAT signaling in breast cancer using TCGA dataset. (B, C) A combined panel of selected breast cancer and near-normal cell lines was reverse-transfected with 10 nM pooled JAK1, JAK2, STAT3 or KRAS siRNAs and cell viability determined after 6 days. Cell viability relative to its own respective control transfected with scramble siRNA was calculated, n = 2–3 (*p < 0.05, p < 0.01). Individual cell lines are shown in panel C. (JPG 2069 kb) [file 13046_2019_1075_MOESM2_ESM.jpg]

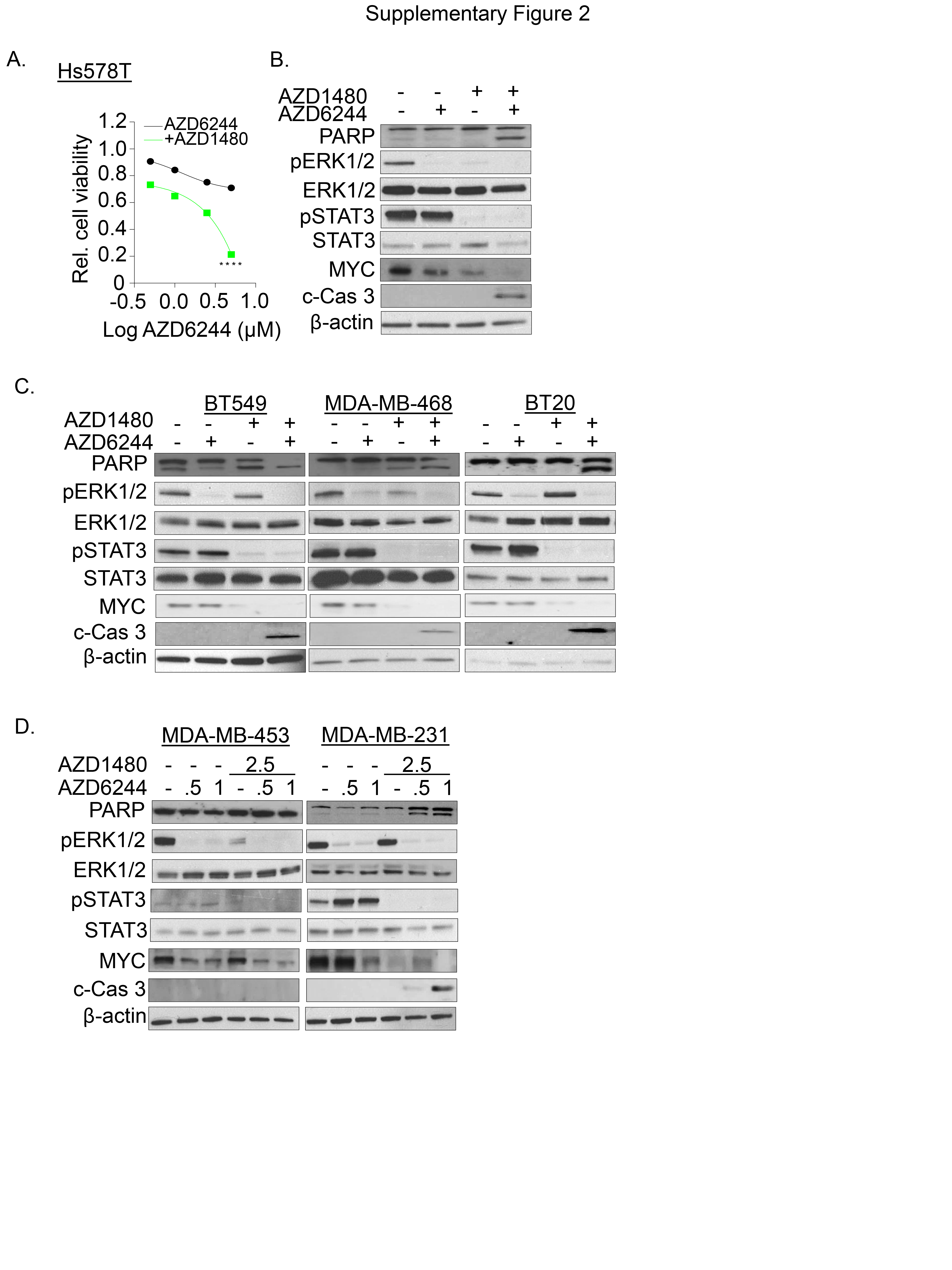

Supplement: Supplementary file 3 — Figure S2: (A) Hs578T cells were exposed to different concentrations of MEK1/2 inhibitor (AZD6244) alone or in combination with JAK2 inhibitor (2.5 μM) and cell viability was determined after 6 days using MTS assays. The dose-response curve was generated by calculating cell viability relative to untreated control and plotted against drug concentration, n = 3 with SEM (***p < 0.001). (B,C, D) Immunoblots analysis in indicated panel of breast cancer cell lines treated with single and combination treatments after 48 h and indicated proteins were determined. (JPG 2427 kb) [file 13046_2019_1075_MOESM3_ESM.jpg]

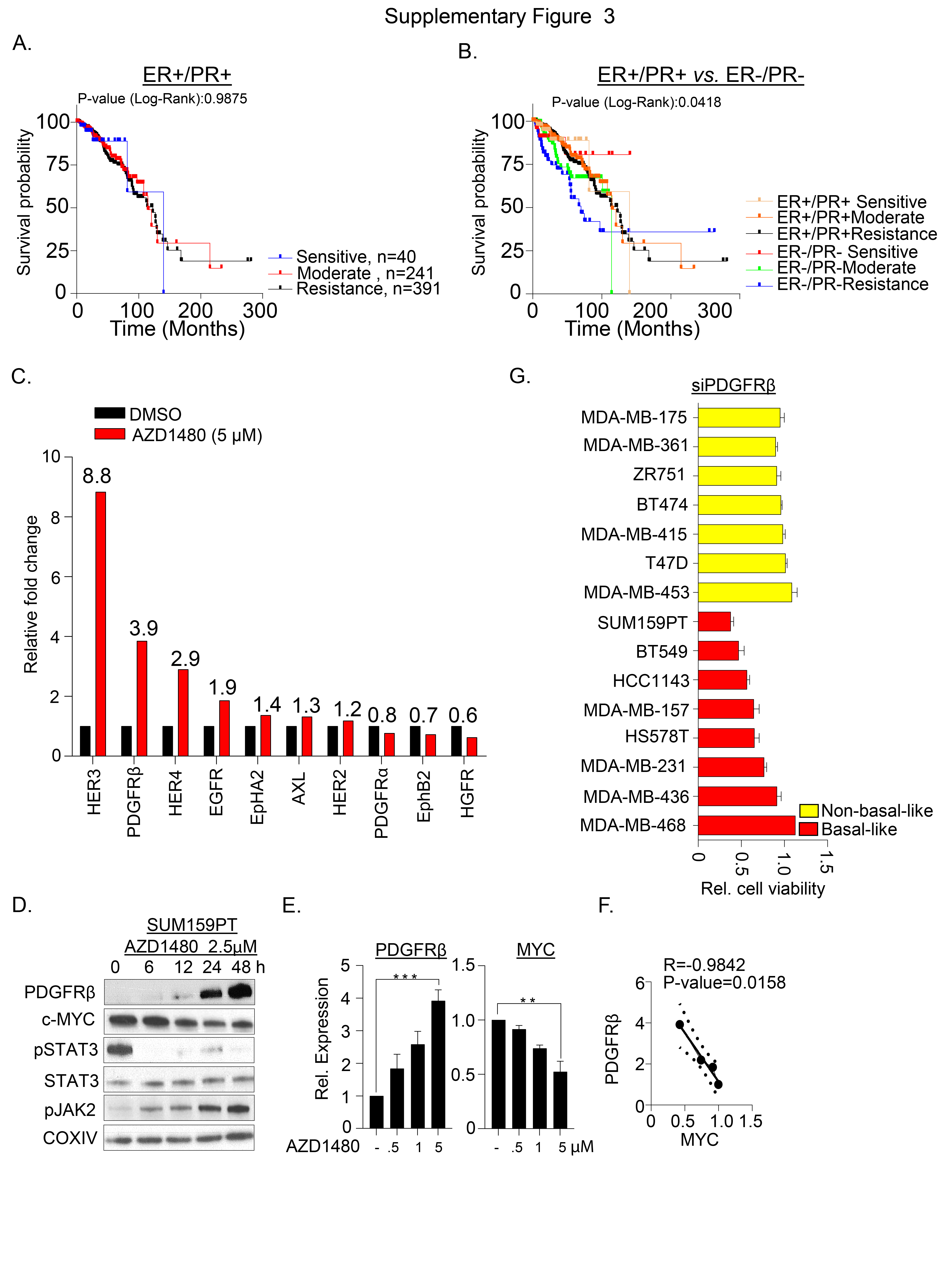

Supplement: Supplementary file 4 — Figure S3: (A,B) Prediction of Kaplan Meier survival analysis in TCGA patients using data derived from cell line treated with Ruxolinitib and computed based on machine-learning (ML) model. See methodology for further details. (C) Relative fold change of phospho-RTK levels derived from Fig. 2c. Quantification of protein-band intensities by densitometric analysis was performed using NIH ImageJ software (NIH, Bethesda, MD). Internal controls of within the array were used to normalize the phosphorylated protein levels. Fold changes are indicated in the graph. (D) SUM159PT cells were treated 2.5 μM AZD1480 as indicated time points and indicated proteins were determined by western blot. (E) Relative fold change of PDGFRβ and MYC mRNA expression upon SUM159PT cells treated with different concentration of AZD1480, n = 3 with SEM (**P ≤ 0.01, ***P ≤ 0.001). (F) Pearson correlation coefficient was calculated between PDGFRB and MYC expression using data derived from panel E. (G) A panel of selected breast cancer and near-normal cell lines was reverse-transfected with 10 nM pooled PDGFRβ siRNAs and cell viability determined after 6 days. Cell viability relative to its own respective control transfected with scramble siRNA was calculated, n = 2–3. (JPG 2271 kb) [file 13046_2019_1075_MOESM4_ESM.jpg]

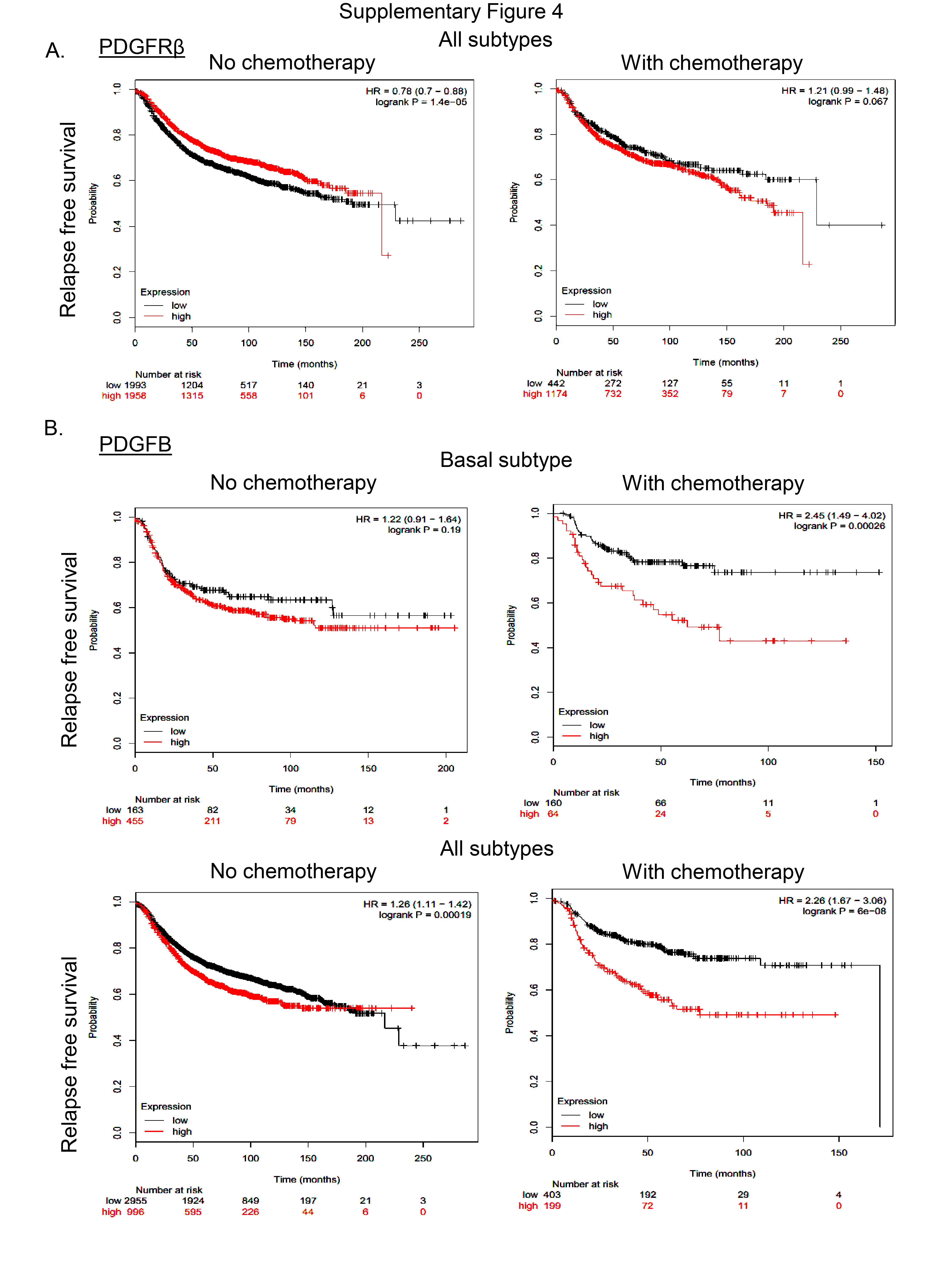

Supplement: Supplementary file 5 — Figure S4: (A, B) Kaplan-Meier survival analysis of the relationship between PDGFRβ or PDGFB mRNA expression and clinical outcomes in breast cancer patients treated with and without chemotherapy using the KMplotter dataset (http://kmplot.com/). PDGFRβ expression stratified on relapse free survival. (JPG 2951 kb) [file 13046_2019_1075_MOESM5_ESM.jpg]

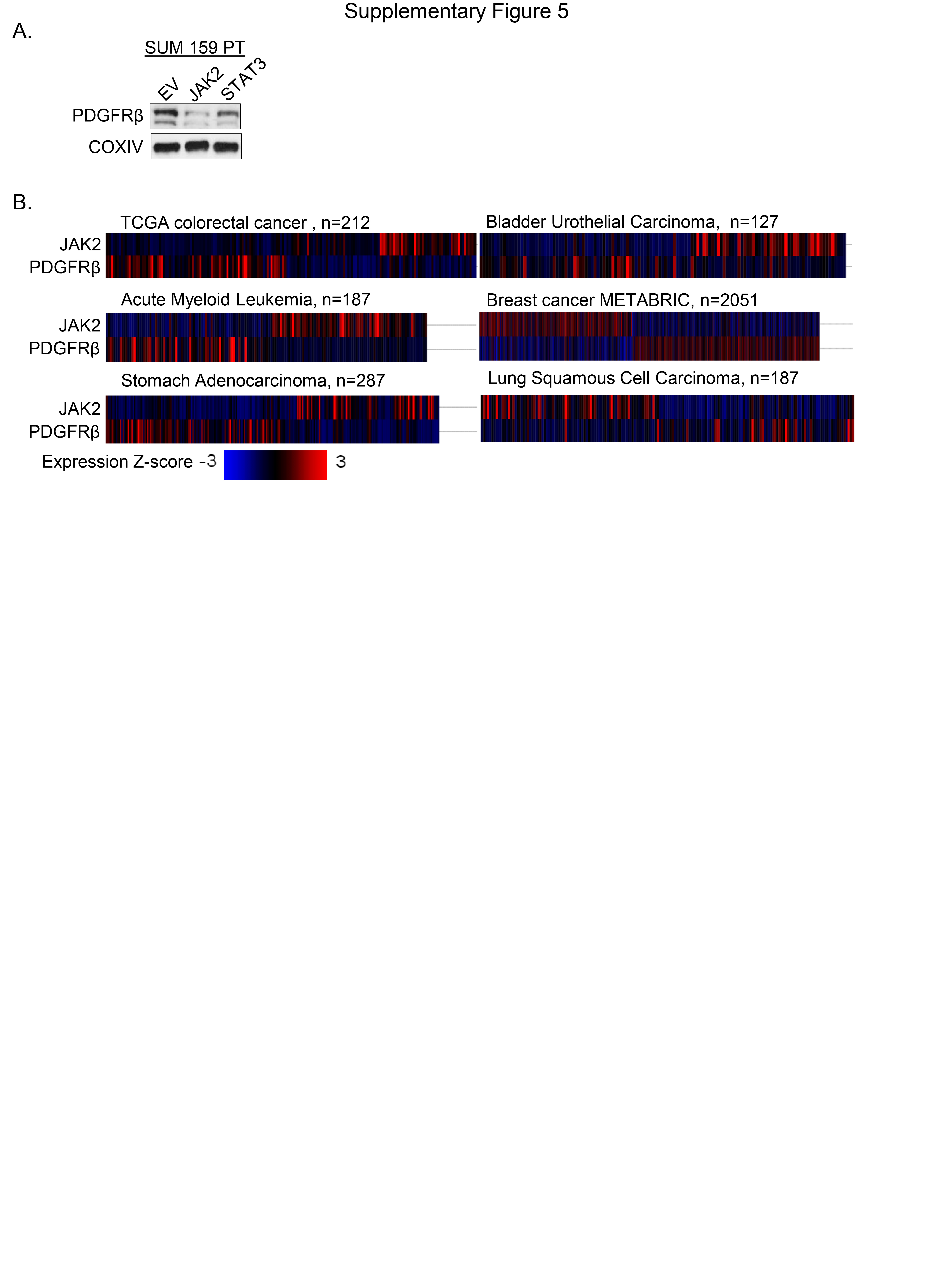

Supplement: Supplementary file 6 — Figure S5: (A) SUM159PT cells were reversed transfected with 1 μg of DNA of empty vector, mJAK2 or STAT3 using Lipofectamine 3000 for 72 h and PDGFRβ levels were determined by western blot. (B) Heatmap analysis of correlation of PDGFRβ with JAK2 levels in pan-TCGA cancer samples. Patient samples were divided into low and high expression. Data derived from cbioportal (http://www.cbioportal.org/). (JPG 1842 kb) [file 13046_2019_1075_MOESM6_ESM.jpg]

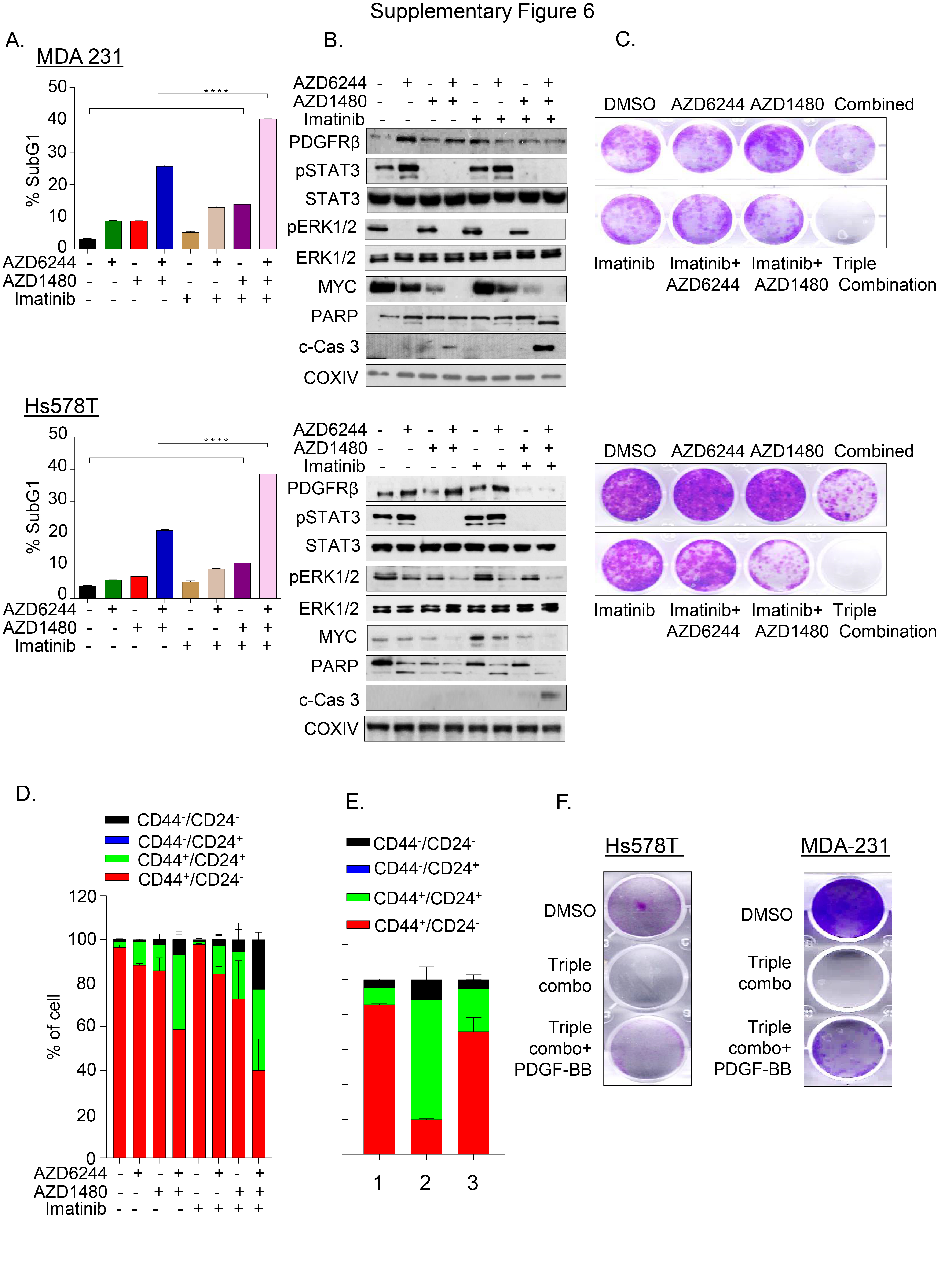

Supplement: Supplementary file 7 — Figure S6: (A) Percentage of sub-G1 population identified using propidium iodide staining and quantified by FACS upon MDA-MB-231 and HS578T cells treated AZD6244 (1 μM), AZD1480 (2.5 μM) and Imatinib (5.0 μM) inhibitors after 72 h, n = 2 with SEM (****p < 0.0001). (B) MDA-MB-231 and HS578T cells treated with indicated concentration of inhibitors as in panel A for 72 h and western blot was performed on to determined levels of indicated proteins. (C) Representative images of colony-forming capacity at 14 days determined using crystal violet staining in MDA-MB-231 and HS578T cells treated indicated concentration of inhibitors as in panel A. (D, E) Quantification of percentage of CD24 and CD44 in Hs578T cells were determined as indicated in Fig. 4e and f, n = 2–3 with SEM. 1: DMSO; 2: Triple-combination; 3: Triple combination with 10 ng/ml of PDGF-BB. (F) Representative Images of colony-forming capacity at 14 days determined using crystal violet staining in SUM159PT cells treated indicated concentration of inhibitors as indicated in panel E and/or stimulated with 10 ng/ml of PDGF-BB ligand. (JPG 3786 kb) [file 13046_2019_1075_MOESM7_ESM.jpg]

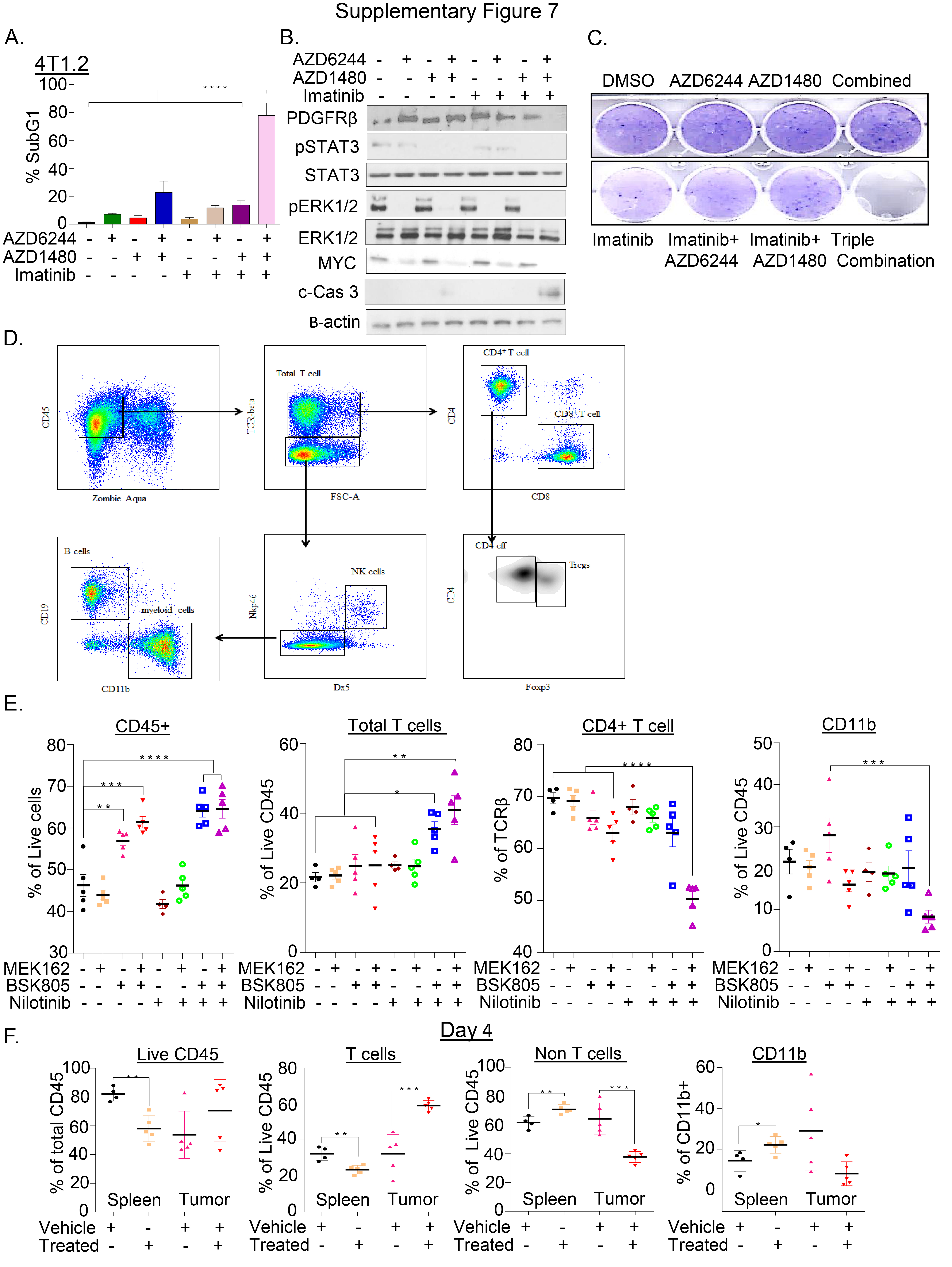

Supplement: Supplementary file 8 — Figure S7: (A) Percentage of sub-G1 population identified using propidium iodide staining and quantified by FACS upon 4 T1.2 cells treated AZD6244 (1 μM), AZD1480 (2.5 μM) and Imatinib (5.0 μM) inhibitors after 72 h, n = 2 with SEM (****p < 0.0001). (B) 4 T1.2 cells treated with indicated concentration of inhibitors as in panel A for 72 h and western blot was performed on to determined levels of indicated proteins. (C) Representative images of colony-forming capacity at 14 days determined using crystal violet staining in 4 T1.2 cells treated indicated concentration of inhibitors as in panel A. (D) Gating strategies to identify subpopulation of immune cells using specific antibodies as indicated within the Fig. (E, F) Percentage of viable immune cells infiltrates gated using indicated antibodies in both spleens and tumor tissues isolated from indicated treatment groups. Graph represents each cell population from six mice/group± SEM (*P ≤ 0.05, **P ≤ 0.01, ***P ≤ 0.001, ****P ≤ 0.0001). (JPG 4558 kb) [file 13046_2019_1075_MOESM8_ESM.jpg]
